# Microbiota-derived metabolites in hematopoietic regulation and disease: mechanisms, context, and therapeutic perspectives

**DOI:** 10.3389/fmicb.2026.1955829

**Published:** 2026-09-24

**Authors:** Husni Farah, Aziz Kubaev, Malathi Hanumanthayya, Maryam Abdul Al-Hussein, Ahmed Faisal Mutee, Fotima Axmedova, Babamuratov Bekzod, Shefali

**Affiliations:** 1 Faculty of Allied Medical Sciences, Hourani Center for Applied Scientific Research, Al-Ahliyya Amman University, Amman, Jordan; 2 Department of Maxillofacial Surgery, Samarkand State Medical University, Samarkand, Uzbekistan; 3 Department of Biotechnology and Genetics, School of Sciences, JAIN (Deemed to be University), Bangalore, Karnataka, India; 4 Department of Medical Laboratory Technologies, College of Medical Technologies, The Islamic University, Najaf, Iraq; 5 College of Pharmacy, Alnoor University, Mosul, Iraq; 6 Urgench Ranch Technology University, Department of Clinical Science, Urgench, Uzbekistan; 7 Department of Medicine, Termez University of Economics and Service, Termez, Uzbekistan; 8 University Institute of Pharma Sciences, Chandigarh University, Mohali, Punjab, India

**Keywords:** erythropoiesis, gut microbiota, hematopoiesis, hematopoietic stem cells, lymphopoiesis, microbial metabolites, myelopoiesis, thrombopoiesis

## Abstract

Recent studies have shown that the gut microbiota plays a crucial role in the regulation of hematopoiesis due to the generation of a variety of metabolites. Metabolites generated by the gut microbiota, including Short-chain fatty acids (SCFAs), tryptophan derivatives, Trimethylamine N-oxide (TMAO), and bile acids, significantly affect the differentiation and function of hematopoietic stem and progenitor cells, as well as immune cell differentiation and activity. The effects of these metabolites are achieved through several signaling pathways, including Histone deacetylase (HDAC) inhibition by SCFAs, aryl hydrocarbon receptor (AHR)-dependent regulation of hematopoietic stem cell fate and myeloid commitment, and inflammasome signaling control by bile acids, among others. Of note, dysregulation of metabolite-mediated pathways has been shown to play a role in the development of several disorders such as anemia, hematologic or solid malignancies, Graft-vs.-host disease (GvHD), and cardiovascular diseases. In this review, we describe current findings on the mechanisms by which microbiota-derived metabolites influence hematopoietic processes and contribute to the development of hematological disorders, while also highlighting novel therapeutic approaches targeting microbiota–metabolite interactions.

## 1 Introduction

Recent research has highlighted the functional interactions between microbial products and host hematopoietic and immune systems, revealing that commensal and pathogenic microbes can shape hematopoiesis and immune regulation through distinct molecular pathways (Manzo and Bhatt, 2015; Fernandez Sanchez et al., 2024). Importantly, microbial metabolites act not only on hematopoietic stem and progenitor cells (HSPCs) but also on the bone marrow niche that includes mesenchymal stem/stromal cells (MSCs), endothelial cells, neurons, and glial cells (Nombela-Arrieta and Isringhausen, 2016; Barman and Goodridge, 2022). These niche elements regulate HSC mobilization, self-renewal, and lineage commitment and can be profoundly influenced by microbial signals such as short-chain fatty acids (SCFAs), lactate, bile acids, and tryptophan derivatives (Khosravi et al., 2014; Manzo and Bhatt, 2015; Pletzer et al., 2017). Thus, the microbiota exerts a dual level of control: directly on HSC biology and indirectly through shaping the supportive stromal microenvironment. Notably, certain commensal bacteria, such as *Clostridium, Faecalibacterium*, and *Lactobacillus*, generate SCFAs, including acetate, propionate, and butyrate, through fermentation of dietary fibers (Hsu et al., 2024). These SCFAs are key regulators of immune cell differentiation and have also been implicated in modulating HSC function via epigenetic and metabolic pathways (Tadokoro and Hirao, 2022). For example, butyrate has been shown to inhibit the proliferation of intestinal stem cells in a Forkhead Box O3 (FoxO3)-dependent manner, suggesting a broader role in stem cell regulation, including within the bone marrow niche (Kaiko et al., 2016). Lactate, another metabolite produced by commensal lactic acid bacteria, stimulates bone marrow MSCs to release stem cell factor (SCF), thereby promoting HSC expansion and hematopoietic regeneration (Lee et al., 2021). This process is mediated via the Gi-coupled protein receptor 81 (GPR81), as evidenced by the significant reduction in bone marrow HSCs in *GPR81*-deficient mice (Ranganathan et al., 2018; Galloway-Peña and Jobin, 2023). In addition to these commensal metabolites, other microbial products, like phosphatidylcholine and γ-glutamylalanine, could be derived from more general microbial sources, like pathobionts, and have been shown to elicit type I interferon (IFN) responses that restore hematopoietic disturbance induced by microbial dysbiosis (Yan et al., 2022; Maknojia et al., 2024). The mechanistic evidence linking microbiota-derived molecules to hematopoietic regulation varies substantially across experimental systems (Zhang et al., 2022). Evidence obtained from human HSPCs or clinical studies provides the strongest basis for inferring relevance to human hematopoiesis, whereas findings from murine models, *ex vivo* systems, or cultured cells should be interpreted as preclinical or mechanistic evidence.

Given the broad effects, dysregulated host–microbiota interactions have been implicated in multiple disease contexts (Grigg and Sonnenberg, 2017; Zheng et al., 2020). Altered microbiota metabolite availability can impair erythropoiesis in anemia, exacerbate graft-vs.-host disease (GVHD), shape the tumor microenvironment in hematologic cancers, and contribute to cardiovascular and inflammatory disorders and infectious diseases (Riwes and Reddy, 2018; Yang and Cong, 2021; Luu et al., 2023; Maciel-Fiuza et al., 2023; Kondapalli et al., 2025; Wang and Wang, 2025). Conversely, restoration of beneficial metabolite pathways through diet, probiotics, or microbial engineering offers promising therapeutic avenues (Abdul Manan, 2025). An important component of microbiota–hematopoiesis communication is the capacity of the host to sense microbial organisms and their molecular signals during both homeostasis and infectious challenge (Kim and Kamada, 2023). Commensal microorganisms provide physiological cues that contribute to immune maturation and hematopoietic homeostasis, whereas pathobionts and infectious agents can generate inflammatory signals that activate innate immune pathways and promote emergency hematopoiesis. Thus, host–microbe and host–pathogen interactions provide an important mechanistic link between microbial ecology, microbial metabolites and products, innate immune sensing, hematopoietic adaptation, and disease. This review discusses the numerous mechanisms by which microbial metabolites influence hematopoietic function and the underlying mechanisms.

## 2 Microbiota, microbiota metabolites, receptors, and functions on the host

The gastrointestinal tract (GIT) harbors a diverse richness of organisms such as bacteria, archaea, fungi, viruses, and bacteriophages referred to as the gut microbiota, an immense ecosystem comprising over 10^15^ microbial cells and more than 22 million microbial genes, surpassing the total number of human cells and genes, and encoding a vast array of enzymes capable of degrading dietary compounds that escape digestion by human enzymes, such as complex polysaccharides and dietary fibers (Figure 1) (Gasaly et al., 2021; Liu et al., 2022). The gut microbiota generates a diverse range of metabolites with extensive biological activities (Postler and Ghosh, 2017). In this review, the term “microbiota-derived metabolites” is used primarily for small molecules whose production or biotransformation is attributable, at least in part, to the gut microbiota (Wang et al., 2026b). These include metabolites directly synthesized by microorganisms, such as SCFAs and several tryptophan-derived compounds, as well as host-derived molecules that are subsequently modified by microbial enzymes, such as secondary bile acids (Wang et al., 2026b). However, not all microbiota-associated bioactive molecules are conventional metabolites. Lactate, and polyamines can originate from both host and microbial metabolism; therefore, their classification as microbiota-derived should be restricted to contexts in which a microbial contribution has been demonstrated or specifically implicated (Yu et al., 2024).

**Figure 1:**
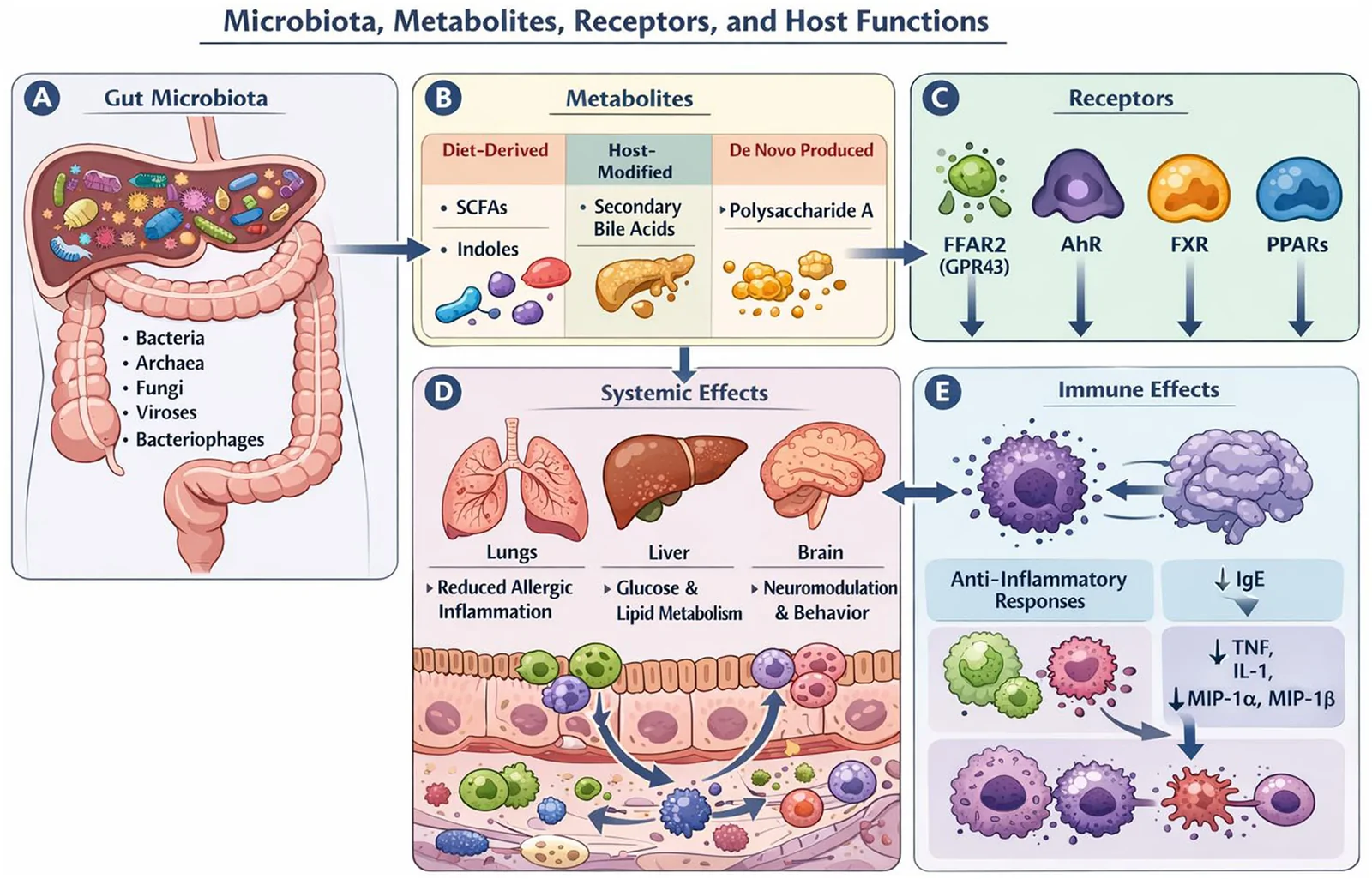
Microbiota-derived metabolites and their receptor-mediated effects on host physiology. **(A)** Gut microbiota composition and metabolic capacity. The gastrointestinal tract harbors a complex microbial ecosystem composed of bacteria, archaea, fungi, viruses, and bacteriophages. This community possesses a vast genetic and enzymatic repertoire that enables the fermentation of dietary fibers, complex polysaccharides, and other substrates that escape host digestion, generating bioactive metabolites with local and systemic effects. **(B)** Microbial and host-modified metabolites. Gut microbiota produce metabolites from dietary components (e.g., short-chain fatty acids (SCFAs) and tryptophan-derived indoles), modify host-derived compounds (e.g., conversion of primary bile acids into secondary bile acids), or synthesize *de novo* products such as polysaccharide A. These metabolites act as energy sources, metabolic intermediates, and signaling molecules. **(C)** Host receptors sensing microbiota-derived metabolites. Microbial metabolites are sensed by host G protein-coupled receptors (GPCRs) and nuclear receptors, including free fatty acid receptor 2 (FFAR2/GPR43), aryl hydrocarbon receptor (AhR), farnesoid X receptor (FXR), and peroxisome proliferator-activated receptors (PPARs). Activation of these receptors initiates intracellular signaling and transcriptional programs regulating immune and metabolic pathways. **(D)** Systemic metabolic and organ-specific effects. Following absorption, metabolites such as SCFAs, bile acids, and indole derivatives enter the circulation and influence distal organs. These effects include reduced allergic inflammation in the lungs, modulation of hepatic glucose and lipid metabolism, enhanced glucagon-like peptide-1 (GLP-1) secretion, improved glucose tolerance, and neuromodulatory effects on the brain. **(E)** Immunomodulatory functions. Microbiota-derived metabolites promote immune homeostasis by enhancing regulatory T cell (Treg) differentiation, increasing anti-inflammatory cytokine production (e.g., IL-10), strengthening epithelial barrier integrity, and suppressing pro-inflammatory mediators such as IgE, TNF, IL-1, MIP-1α, and MIP-1β. Collectively, these mechanisms contribute to balanced mucosal and systemic immune responses.

Microbial metabolites are generally believed to have two main functions: serving as “fuels” or energy sources and acting as fundamental elements in metabolic processes (Zhang et al., 2023). Nevertheless, it has been demonstrated that certain metabolites can function as intracellular signaling molecules by regulating the activity of enzymes, such as histone deacetylases (HDACs), inhibiting enzymes in traditional feedback loops of metabolic pathways, or directly acting as signaling molecules by binding to nuclear hormone receptors, such as peroxisome proliferator-activated receptor (PPAR) and farnesoid X receptor (FXR) (also known as bile acid receptor) (Zhang et al., 2023). It is widely recognized that some microbiota metabolites have a strong effect on the generation of hormones and stimulation of the GIT by activating certain G protein-coupled receptors (GPCRs) (Melhem et al., 2019). Currently, as orphan GPCRs were being gradually identified and connected with specific chemicals, it became clear that several other compounds may be recognized and linked by selective GPCRs to initiate traditional signal transduction pathways (Paul et al., 2026). For instance, the gut microbiota produces SCFAs and secondary bile acids (SBAs), whereas the host's intermediate metabolism produces circulatory metabolites such as lactate and ketone bodies (Zhang et al., 2023). These compounds are referred to as “signaling metabolites” (Zhang et al., 2023).

SCFAs have a role in maintaining immune balance in both the mucosal and systemic parts of the body (McCarville et al., 2020). SCFAs promote a state of balanced immunological response in the colon by activating regulatory T cells (Tregs) through a process that relies on the free fatty acid receptor 2 (FFAR2) (also known as GPR43) (Carretta et al., 2021). These interactions have a crucial role in the generation of the anti-inflammatory cytokine Interleukin (IL)-10 and in protecting against colitis in mouse models (McCarville et al., 2020). The anti-inflammatory effects of SCFAs have also been associated with the activation of the inflammasome in the cells that line the intestines (Carretta et al., 2021). Indoles, tryptamine, serotonin, and kynurenine are among the well-known substances that are produced when tryptophan is broken down (Hubková et al., 2022). Dietary tryptophan can create indoles derived from microbes, such as indole-propionic acid, indole-3-acetate, indole-3-aldehyde, tryptamine, and 3-methyl-indole (Li X. et al., 2021). These metabolites affect immunity, metabolism, behavior, and lifespan, in addition to other aspects of the host, through their interaction with the nuclear receptor known as the aryl hydrocarbon receptor (AhR). Bile acids impact the microbiota, which in turn affects host metabolism (He et al., 2025). In mice, activation of FXR by the fexaramine agonist modifies the makeup of the gut microbiota and raises serum glucagon-like peptide 1 (GLP-1) and glucose tolerance (McCarville et al., 2020). Recent research has found a connection between bile acids and gut bacteria in the pathogenesis of colorectal cancer (CRC) (Keane et al., 2023). Furthermore, spermine inhibits the generation of IL-1, macrophage inflammatory protein-1 (MIP-1) α, and MIP-1β in human peripheral blood mononuclear cells (Zhang et al., 1997). Moreover, the development of intestinal and systemic immune cells is impacted by the daily treatment of either spermine or spermidine in rats from birth (Pérez-Cano et al., 2010). In summary, the microbiota, their metabolites, receptors, and host activities interact dynamically to form a complex ecosystem that is precisely regulated inside the human body.

## 3 Methodology for the literature review

The present review was conducted to comprehensively summarize the current evidence regarding microbial metabolites in hematopoietic regulation and disease. To ensure broad coverage of the available literature, a systematic bibliographic search was performed using the Web of Science, PubMed, and Google Scholar databases. The primary search keywords included metabolites generated by the gut microbiota, including SCFAs, tryptophan derivatives, TMAO, and bile acids, which have a considerable effect on the differentiation and functioning of hematopoietic stem and progenitor cells as well as immune cell differentiation and activity. The reference lists of relevant publications were also carefully reviewed to identify additional studies that may not have been captured in the initial database search. No publication-year restrictions were applied, allowing us to include both foundational studies and recent advances in the field. English-language articles were preferentially considered, and both preclinical and clinical studies were included. Study selection was conducted in two stages: titles and abstracts were first screened for relevance, followed by a thorough full-text assessment of potentially eligible articles.

## 4 Microbial metabolites and hematopoietic niches: from fetal development to adult adaptation

Microbial signals in early life are critical to guide the development of HSPCs in bone marrow (Liu et al., 2024). Maternal microbiome metabolites have been reported to pass through the placenta and into fetal circulation, which impacts fetal hematopoietic development (Tamburini et al., 2016). *In vivo* experiments have also shown that microbial communities control the development of HSCs and HSPCs by modulating inflammatory signaling in their niche (Zhong et al., 2023). A balanced maternal microbiome is thus essential to modulate normal embryonic hematopoiesis and HSPC homeostasis (Liu et al., 2024). Specific bacterial lineages were found to influence the regulation of the differentiation of HSPCs by modulating the production of neighboring cytokines around these progenitors (Zhong et al., 2023). Microbial metabolites, such as SCFAs, are also passed to the offspring via breast milk, where they influence the adjustment of immune maturation and inflammatory homeostasis (Smith et al., 2013). In addition to this, the gut MAMPs get into systemic circulation and travel to the bone marrow, where they suppress the proliferation and lineage commitment of HSCs, multipotent progenitors (MPPs), and their derivatives (Balmer et al., 2014). Interruptions of maternal microbiota, e.g., dysbiosis, could hinder the process and make the neonatal bone marrow microenvironment different, thereby influencing the activity of progenitor cells (Lu et al., 2024). Collectively, these discoveries shed light on how maternal microbiota signals control the immune system and hematopoietic development in early life (McCoy and Thomson, 2018).

Systemic metabolites can either potentiate or suppress these emergency responses by acting directly on HSPCs or indirectly via niche cells and inflammatory mediators (Barman and Goodridge, 2022; Wang et al., 2025). For instance, SCFAs are present in trace amounts in fetal direct circulation, but maternal nutrition and placental transfer can transfer SCFAs to the embryo (Qin et al., 2025). SCFAs in adults regulate bone marrow HSCs indirectly by modulating stromal cytokine secretion and systemic inflammation (Liu et al., 2024). Propionate and butyrate are capable of inhibiting inflammatory signaling and altering the balance in myelopoiesis vs. lymphopoiesis; propionate was implicated in influencing IL-17–producing innate T cell responses and in HDAC-dependent modifications of innate immune ontogeny, secondarily modulating HSC output (Nastasi et al., 2017; Porbahaie et al., 2023). SCFAs are thus able to inhibit unwarranted emergency myelopoiesis or, in a context-dependent fashion, facilitate lineage differentiation. Concentrations of fetal circulation bile acids are unique compared with adults; pathologic exposure (maternal cholestasis, bile acid dysregulation of metabolism) can interfere with fetal hematopoiesis via placental inflammation and oxidative stress (Colombo et al., 1985). Microbiota-derived second bile acids can gain systemic circulation and modulate marrow stromal function via FXR/G protein-coupled receptor 5 (TGR5) on niche cells and induce epithelial barrier breakdown and low-grade systemic inflammation that initiates emergency myelopoiesis (Song et al., 2025). Deoxycholic acid (DCA) specifically has been implicated in secondary reprogramming of hematopoiesis through epithelial stress and inflammation (Ruutu et al., 2002; Tay et al., 2007). In summary, microbial metabolites offer systemic signals that influence hematopoiesis at every phase of life, from fetal ontogeny to adult immune competence (Figure 2).

**Figure 2:**
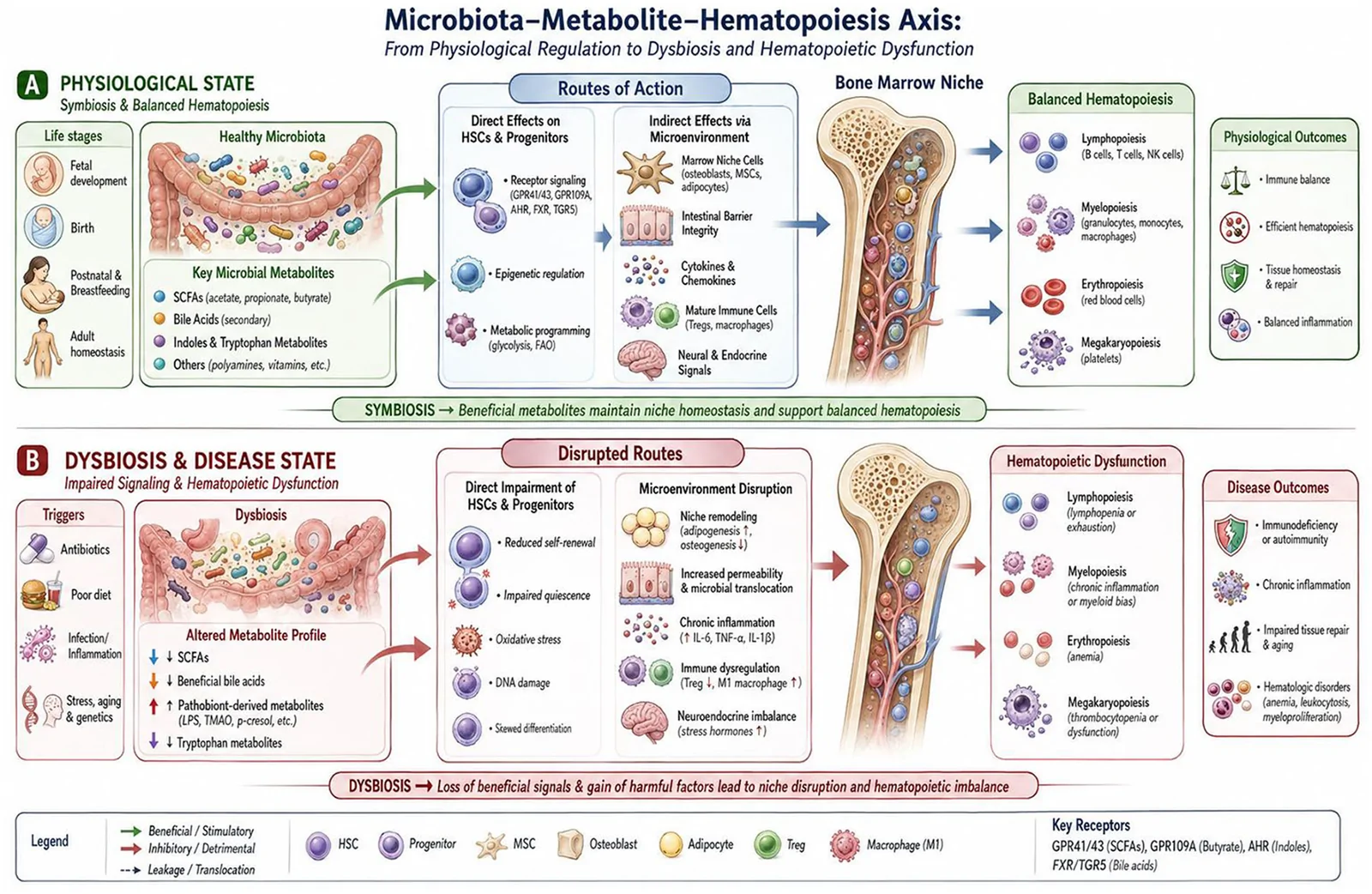
Microbiota–metabolite–hematopoiesis axis: from physiological regulation to dysbiosis-associated hematopoietic dysfunction. The schematic presents a conceptual framework distinguishing the beneficial effects of microbiota-derived metabolites under physiological conditions from the disrupted signaling associated with gut dysbiosis and disease. **(A)** Physiological state: During fetal development, birth, breastfeeding, and adulthood, a balanced microbiota generates bioactive metabolites, including short-chain fatty acids (SCFAs), bile acids, indole/tryptophan-derived metabolites, polyamines, and vitamins. These metabolites influence hematopoietic stem and progenitor cells (HSPCs) through direct mechanisms, including receptor-mediated signaling, epigenetic regulation, and metabolic programming, and through indirect mechanisms involving mesenchymal stromal cells, osteoblasts, adipocytes, intestinal barrier integrity, cytokines/chemokines, mature immune cells, and neural/endocrine signaling. Coordinated communication between these compartments preserves bone marrow niche homeostasis and supports balanced lymphopoiesis, myelopoiesis, erythropoiesis, and megakaryopoiesis. **(B)** Dysbiosis and disease state: Antibiotic exposure, poor diet, infection/inflammation, stress, aging, and genetic factors can disrupt microbial community structure and alter the metabolite profile, including reduced beneficial SCFAs and bile acid signaling and increased potentially pathogenic microbial products. These changes may directly impair HSPC self-renewal, quiescence, survival, and differentiation while indirectly disrupting the marrow microenvironment through niche remodeling, increased intestinal permeability and microbial translocation, chronic inflammation, immune dysregulation, and altered neuroendocrine signaling. Consequently, dysbiosis-associated signaling imbalance can promote abnormal lymphopoiesis, myelopoiesis, erythropoiesis, and megakaryopoiesis, ultimately contributing to immunodeficiency or autoimmunity, chronic inflammation, impaired tissue repair, aging-associated dysfunction, and hematological disorders. Green arrows indicate beneficial/stimulatory signaling, red arrows indicate inhibitory/detrimental effects, and dashed arrows indicate leakage or translocation. Key metabolite-sensing pathways include GPR41/GPR43, GPR109A, AHR, FXR, and TGR5.

## 5 Microbial metabolites and their impact on resident immune cells across tissue barriers and metabolic organs

The human microbiota generates a vast array of metabolites that cross the gut lumen and are vital regulators of host immunity (Rooks and Garrett, 2016). These metabolites control immune homeostasis not merely at the intestinal mucosa but at distant tissue barriers and organs such as the skin, lungs, liver, adipose tissue, and the CNS (Liu et al., 2022). Tissue-resident immune cells like dendritic cells (DCs), macrophages, innate lymphoid cells (ILCs), γδ T cells, and regulatory T cells are especially responsive to these microbial signals (Kang et al., 2020; Seo et al., 2020; Wang et al., 2023; Mohamed et al., 2024; Shvets et al., 2024). Through regulation of their function, microbial metabolites are involved in immune tolerance, pathogen defense, and inflammation modulation.

The gut is a dense microbial community and a metabolic crosstalk hot spot (Krishnamurthy et al., 2023). Diet fiber fermentation releases SCFAs (acetate, propionate, and butyrate), promoting epithelial barrier function and regulating differentiation of regulatory T cells by epigenetic reprogramming and GPCR activation (Tan et al., 2023). SCFAs also control DC and macrophage pro-inflammatory responses and induce a tolerogenic state (Kim, 2021). Other metabolites provide further layers of regulation. Secondary bile acids, produced by microbial modification of primary bile acids, suppress T cell helper (Th) 17 reactions and modulate intraepithelial lymphocyte homeostasis (Hang et al., 2019). In contrast, tryptophan catabolites are strong agonists for the AhR, enabling ILC3-mediated IL-22 production, which induces mucosal protection (Zelante et al., 2013; Watanabe-Asaka et al., 2021).

The skin microbiota adds its own set of metabolites to the portfolio, such as indole derivatives, lipids, and SCFA-like molecules (Jiminez and Yusuf, 2023; Smythe and Wilkinson, 2023). Indoles, the tryptophan metabolites of bacteria, activate AhR in keratinocytes and cutaneous T cells and increase antimicrobial peptide expression (Jiminez and Yusuf, 2023). Microbial lipids reset skin-resident macrophage and Langerhans cell function, influencing wound healing and inflammatory thresholds (Lunjani et al., 2021; Lee and Sacks, 2024). Commensal bacteria-derived metabolites, at the same time, prime epidermal γδ T cells to facilitate a rapid tissue repair response after injury (Hu et al., 2022; Yang et al., 2024). Besides, in spite of reduced microbial burden of the respiratory tract, immune control remains under the influence of local microbes and gut-derived metabolites transported via the circulation (De Córdoba-Ansón et al., 2025). SCFAs, for example, augment alveolar macrophage phagocytic and immunomodulatory activity and suppress allergic and inflammatory reactions (Williams et al., 2022). Aberrations in this metabolic signaling—always present with dysbiosis—have been associated with asthma and susceptibility to infection (Kim et al., 2024).

Located downstream of the gut via the portal vein, the liver is constantly exposed to microbial metabolites (Brandl et al., 2017). Secondary bile acids are also important in regulating Kupffer cell activation and hepatocyte signaling via FXR and TGR5, shaping the organ's inflammatory phenotype (Chiang and Ferrell, 2020). Microbial products like acetate and ethanol also modulate hepatocyte metabolism and innate immune tone (Zhou and Fan, 2019). Perturbation of these interactions forms the underpinning for diseases like non-alcoholic fatty liver disease (NAFLD) and hepatocellular carcinoma (HCC) (Dua et al., 2025). Additionally, adipose depots are currently recognized to be immunologically active organs where microbial metabolites have systemic actions (Mauney et al., 2025). SCFAs induce anti-inflammatory macrophage polarization and regulatory T cell accumulation, guaranteeing metabolic homeostasis (May and Den Hartigh, 2023). In the end, microbial metabolites are pivotal in two-way communication along the gut–brain axis (Suganya and Koo, 2020). In the CNS, microbial metabolites have profound influences on resident immune cells. SCFAs trigger maturation and functional calibration of microglia to enable maximum surveillance and synaptic support (Mirzaei et al., 2021b). Tryptophan-derived ligands, by activating AhR in astrocytes and microglia, inhibit neuroinflammation, a mechanism implicated in multiple sclerosis and its related disorders (Rothhammer et al., 2016). Lactate, a product of microbial fermentation, is neuroprotective through the strengthening of regulatory immune cycles in the CNS (Wu et al., 2023). When the production of metabolites is changed, however, these beneficial interactions are disrupted, making one susceptible to psychiatric and neurodegenerative illness (Missiego-Beltrán and Beltrán-Velasco, 2024). In total, microbial metabolites are promiscuous molecular signals that repeatedly report to resident immune cells of barrier surfaces, metabolic organs, and the nervous system. Elucidating these interactions not only enhances our appreciation for host–microbe symbiosis but also promises new therapies that exploit microbial metabolism to restore immune balance across the body.

## 6 Microbial metabolites on hematopoiesis

The interplay of microbial metabolites and hematopoiesis represents a key axis of host-microbe communication, influencing blood cell development via direct effects on HSCs and the bone marrow microenvironment (Agarwal et al., 2025). These interactions are shaped not only by the type of metabolite but also by the taxonomic identity of the microbial producers—whether commensals, pathobionts, or overt pathogens (Liu et al., 2024). This section outlines the role of specific microbial metabolites in regulating hematopoiesis, focusing on erythropoiesis as a representative lineage (Figure 3, Table 1).

**Figure 3:**
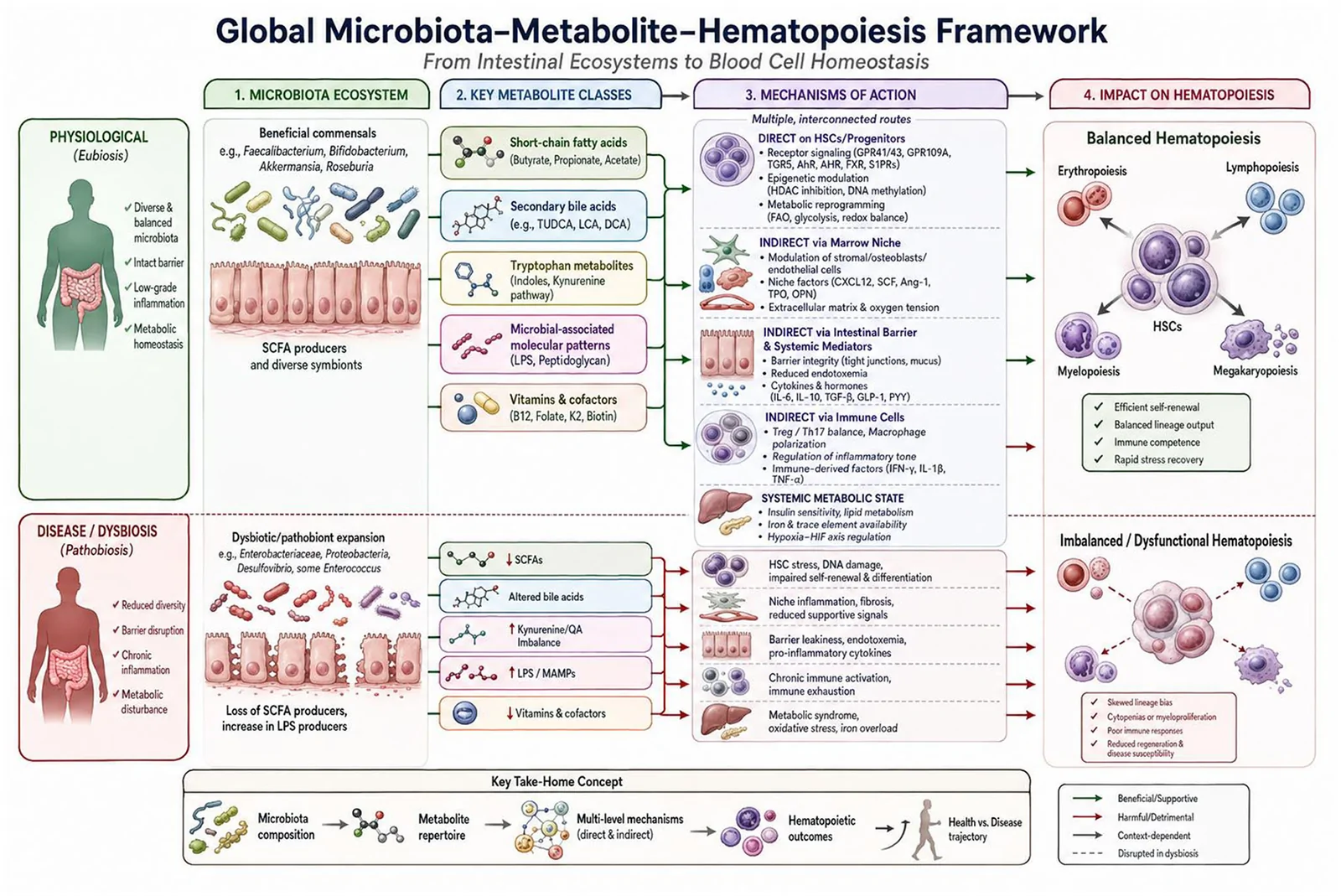
Global microbiota–metabolite–hematopoiesis framework linking intestinal homeostasis to blood-cell production. The figure proposes a conceptual framework illustrating how gut microbiota composition and the microbial metabolite repertoire collectively regulate hematopoiesis through both direct and indirect mechanisms, while emphasizing the contrast between physiological eubiosis and disease-associated dysbiosis. Under physiological conditions, a diverse and metabolically balanced microbiota, together with an intact intestinal barrier, generates beneficial metabolites including short-chain fatty acids (SCFAs; butyrate, propionate, and acetate), secondary bile acids, tryptophan-derived metabolites, vitamins, and other microbial signals. These factors can act directly on hematopoietic stem and progenitor cells (HSPCs) through receptor-mediated signaling, metabolic reprogramming, and epigenetic mechanisms, or indirectly by modifying the bone-marrow niche, intestinal barrier integrity, systemic cytokine and hormonal profiles, and mature immune-cell function. The direct arm represents metabolite-dependent signaling in HSCs and progenitors through receptors and intracellular pathways, including GPR41/43, GPR109A, TGR5, aryl hydrocarbon receptor (AhR), and other metabolite-responsive pathways, as well as epigenetic regulation by histone deacetylase inhibition and altered cellular metabolism. The niche-mediated arm illustrates effects on stromal, endothelial, osteoblastic, and other marrow-resident cells, including modulation of niche factors such as CXCL12, SCF, ANG-1, TPO, and oxygen tension. The intestinal/systemic arm highlights preservation of epithelial integrity, mucus and tight-junction function, limitation of endotoxin translocation, and regulation of circulating cytokines and metabolic hormones. In parallel, microbial metabolites influence mature immune cells, including Treg/Th17 balance, macrophage polarization, and inflammatory mediator production, thereby creating additional feedback between immunity and hematopoiesis. In contrast, microbiota dysbiosis is characterized by reduced microbial diversity, loss of SCFA-producing organisms, expansion of potentially pathogenic taxa, altered bile-acid and tryptophan metabolism, increased microbial-associated molecular patterns (e.g., LPS), and impaired production of vitamins and cofactors. These changes can disrupt the intestinal barrier and promote systemic inflammation, endotoxemia, metabolic stress, and inflammatory remodeling of the bone-marrow niche. Consequently, HSCs and progenitors may undergo cellular stress, altered self-renewal and differentiation, and lineage bias, ultimately contributing to dysfunctional erythropoiesis, lymphopoiesis, myelopoiesis, and megakaryopoiesis.

**Table 1:** Role of microbiota-derived metabolites in hematopoiesis.

Metabolite	Study setting	Process	Mechanism	Description	References
Acetate, propionate, and butyrate	*In vivo*	Erythropoiesis	*g**-**globin* gene expression	Short-chain fatty acids (SCFAs) stimulate the activation of embryonic globin genes in erythroid fetal liver cells (eFLCs).	Bhatia et al., 2009
Acetate	*In vivo*	Erythropoiesis	hypoxia-inducible factor-2 (HIF-2)	Acetate served as a signaling chemical employed by a particular acetyl-CoA generator, ACSS2, to control the relationships between HIF-2α and its specific acetyltransferase and coactivator, CBP.	Xu et al., 2014
Butyrate	*In vivo*	Hematopoietic stem cells (HSCs) self-renewal and differentiation	Iron access	The microbiota's butyrate regulates the self-renewal and differentiation of HSCs in the bone marrow during stressful conditions by influencing the availability of iron in that area.	Zhang et al., 2022
Butyrate	*In vitro*	Homeostasis	NLR family pyrin domain containing 3 (NLRP3)	The study provided evidence that butyrate efficiently inhibits the activation of the NLRP3 inflammasome.	Cortez et al., 2022
Acetate, propionate, and butyrate	*In vitro* and *in vivo*	Chemotaxis and migration	CINC-2αβ	SCFAs stimulated the movement of neutrophils, which occurred at the same time as an increase in levels of the CINC-2αβ chemoattractant and an enhanced expression of L-selectin on the surface of neutrophils.	Vinolo et al., 2009
Butyrate and acetate	*In vitro* and *in vivo*	Antimicrobial phenotype	Differentiation	The production of SCFAs by bacteria stimulates macrophages to exhibit an increased antimicrobial response, hence improving their capacity to efficiently remove intracellular infections.	Jukes et al., 2020
Butyrate	*In vitro* and *in vivo*	Differentiation	Blimp-1 and Xbp-1	This study has shown that epigenetic mechanisms control the production of an enzyme called AID, as well as transcription factors Blimp-1 and Xbp-1. These molecules play a crucial role in the development of B cells.	White et al., 2014
Acetate, butyrate, and propionate	*In vitro* and *in vivo*	Metabolism	Oxidative phosphorylation, glycolysis, and fatty acid synthesis	SCFAs increase the amounts of acetyl-CoA in B cells and regulate metabolic sensors to enhance the processes of fatty acid synthesis, oxidative phosphorylation, and glycolysis.	Kim et al., 2016
Butyrate	*In vitro*	Natural killer (NK) cell	Mammalian target of rapamycin complex 1 (mTORC1), c-Myc	Butyrate limited the functionality of NK cells by reducing the activity of mTORC1, the production of c-Myc mRNA, and metabolic processes.	Zaiatz-Bittencourt et al., 2023
Butyrate	*In vivo*	Liver-resident natural killer cell homeostasis	Interleukin (IL)-18/IL-18R signaling	The results reveal a regulatory network of the gut-liver axis, emphasizing the significance of the microbiota in early life for the formation of immune cells in the tissues.	Tian et al., 2023
3-indopropionic acid (IPA)	*In vitro* and *in vivo*	γδT cells	-	The gut microbiota has an impact on the antitumor capabilities of γδT cells and has identified putative intermediary chemicals that establish a connection between the gut microbiota and γδT cells.	(Han et al., 2020)
Phosphatidylcholine and γ-glutamylalanine	*In vivo*	Hematopoiesis	Type I interferon (IFN) response	Signal Transducer and Activator of Transcription 1 (STAT1) signaling in non-myeloid hematopoietic cells was activated by the microbiota to support hematopoiesis, and type I IFNs were specifically required for this signaling.	Yan et al., 2022
Trimethylamine N-oxide (TMAO)	*In vivo*	Platelet hyperreactivity and thrombosis	Release of Ca2+	Platelets exposed directly to TMAO experienced increased submaximal stimulus-dependent platelet activation from multiple agonists due to greater calcium release from intracellular reserves.	Zhu et al., 2016
TMAO	*In vivo*	Platelet activation	-	The results show that TMAO did not increase platelet responsiveness under the experimental conditions.	Emonds et al., 2022
Lactate	*In vivo*	Erythropoiesis	Stem cell factor (SCF)- G-protein-coupled receptor (GPR) 81	Lactate from the microbiota increases SCF production in LepR+ Bone marrow (BM) Mesenchymal stem cells (MSCs), which drives hematopoiesis and erythropoiesis in a GPR81-dependent way.	Lee et al., 2021
Cholic acid (CA), ursodeoxycholic acid (UDCA), deoxycholic acid (DCA), hyocholic acid (HCA), chenodeoxycholic acid (CDCA), alpha-muricholic acid (α-MCA), beta-muricholic acid (β-MCA), omega-muricholic acid (ω-MCA), murocholic acid (Muro CA), dehydrocholic acid (DHCA), hyodeoxycholic acid (HDCA), taurocholic acid (TCA), TUDCA, tauro-alpha-muricholic acid (T-α-MCA), tauro-beta-muricholicacid (T-β-MCA), tauro-omega-muricholic acid (T-ω-MCA), taurodeoxycholic acid (TDCA), taurochenodeoxycholic acid (TCDCA), taurodehydrocholic acid (TDHCA), taurohyocholic acid (THCA), taurohyodeoxycholic acid (THDCA), glychocolic acid (GCA), glycoursodeoxycholic acid (GUDCA), glycochenodeoxycholic acid (GCDCA), glycodeoxycholic acid (GDCA), glycodehydrocholic acid (GDHCA), glycohyocholic acid (GHCA), and glycolithocholic acid (GLCA)	*In vivo*	Erythropoiesis	Endoplasmic reticulum (ER) stress	Implanting TBA-preconditioned hematopoietic stem and progenitor cells (HSPCs) that express ENT3 into mice lacking the Slc29a3 gene enhances the ability of the bone marrow to regenerate and increases the amount of the red blood cell population, hence preventing premature deaths. To summarize, the present study reveals that ENT3 plays a crucial function in maintaining the balance of blood cell production by transporting lysosomal bile acid in a backward direction, which is necessary for normal growth of red blood cells in mice.	Persaud et al., 2021
DCA and lithocholic acid (LCA)	*In vivo*	Myelopoiesis	Colony-forming unit–granulocyte and macrophage (CFU-GM); granulocyte-monocyte progenitors (GMPs)- vitamin D receptor (VDR)	The effect of DCA on HSPCs, leading to the expansion of GMPs in a VDR-dependent way, indicates that interactions between the microbiota and the host could directly impact hematopoiesis and potentially influence the severity of infectious and inflammatory diseases.	Thompson et al., 2023
Bile acids	*In vivo*	Bone marrow homeostasis	Takeda-G protein-receptor-5 (TGR5)	The increase of adipocyte precursors and the concurrent loss of BMAT in TGR5-deficient animals indicate that TGR5 is required for the *in vivo* maturation of bone marrow adipocytes in young male mice.	Alonso-Calleja et al., 2023
TCA and glycolic acid (GCA)	*In vivo* and *in vitro*	anti-inflammatory	Farnesoid X Receptor (FXR)	The bile acids effectively suppressed the recruitment of macrophages induced by lipopolysaccharide (LPS) and the release of proinflammatory cytokines/chemokines.	Ge et al., 2023
TCA and TUDCA	*In vitro* and *in vivo*	Fetal hematopoiesis	ER stress	TCA, which is the main bile acid in a fetus's liver, helps HSCs grow in a lab setting by stopping proteins from sticking together. Lowering bile acid levels in living things causes ER stress to rise and proteins to aggregate, which greatly reduces the number of fetal liver HSCs.	Sigurdsson et al., 2016

### 6.1 Erythropoiesis and red blood cells

In contrast to pathogen-derived products, commensal-derived microbial metabolites such as butyrate, propionate, and acetate exert more homeostatic effects on erythropoiesis. In a study, Bhatia et al. (2009) conducted a study to assess the impact of SCFAs on erythropoiesis in both primitive and definitive erythroid precursor cells. These cells were obtained from transgenic mice with naturally high levels of SCFAs, as well as from wild-type mice and transgenic mice that contained the human β*-globin* gene locus. From a mechanistic standpoint, it has been shown that SCFAs have an impact on several intracellular processes and regulatory molecules (Bhatia et al., 2009). These include cyclic nucleotides, cell-signaling kinases, transcription factors, mediators of apoptosis, corepressors, and histone deacetylase inhibitors (Bhatia et al., 2009). The activation of signaling molecules by SCFA has been linked to erythropoiesis and the increase in expression of embryonic and fetal globin genes (Mabaera et al., 2008). Bhatia et al. (2009) indicated that SCFAs stimulate the activation of embryonic and fetal β-type *globin* genes in EryD, which is a crucial objective in the treatment of β*-globin* gene abnormalities. This discovery provides the chance to examine SCFAs in a model of definitive erythropoiesis that is sensitive to SCFAs, not altered, and can be easily modified by transgenic modification. The current study investigated the impact of naturally occurring increases in SCFAs on the expression of globin genes in immature red blood cell precursors (Bhatia et al., 2009). This might elucidate the difference in induced human fetal (gA vs. gG) or induced murine embryonic (bH1 vs. eY) globin gene expression found with SCFAs alone vs. SCFAs with ins/erythropoietin (Bhatia et al., 2009). Collectively, these results highlight the prime significance of the microbial origin of the metabolites—commensal or pathogenic—since such a differentiation is responsible for making the metabolite foster erythroid development or compromising it through stimulation.

### 6.2 Lymphocytes

The most significant anti-inflammatory characteristic of SCFAs is their capacity to stimulate Tregs, which in turn reduces the activity of effector T cells. This impact is mediated by a variety of mechanisms: acetate and propionate, but not butyrate, boost the growth of pre-existing colonic Tregs (cTregs), while propionate and butyrate, but not acetate, promote the *de novo* differentiation of naïve T cells into Tregs (Arpaia et al., 2013; Smith et al., 2013). Each of these roles works independently since the number of Tregs in germ-free mice, which is drastically decreased in the absence of therapy, may be restored by separately administering butyrate, acetate, or propionate (Arpaia et al., 2013; Smith et al., 2013). The particular nature of each action for a distinct group of SCFAs reflects the underlying biochemical mechanism. Butyrate does not efficiently activate FFAR2, which is necessary to proliferate pre-existing cTregs (Smith et al., 2013). Separately, butyrate has been demonstrated to increase Treg production by fostering an anti-inflammatory phenotype in macrophages and DCs via HCAR2 activation (Singh N. et al., 2014). In addition to these mechanisms, Atarashi et al. (2013) discovered that exposing a CRC cell line to SCFAs may increase the generation of transforming growth factor beta (TGF-β), facilitating the differentiation of naïve T cells into Tregs. SCFAs have been disclosed to encourage the generation of both effector and Tregs based on the immune-mediated setting (Park et al., 2015). B-cell function is also modulated by commensal-derived SCFAs (Kudoh et al., 1998). In a study, Kim et al. (2016) assessed this discovery further, demonstrating that SCFAs boost antibody responses by modulating gene expression via HDAC inhibition. Intriguingly, they discovered that SCFAs affect the energy metabolism of B cells, promoting antibody synthesis.

Recent investigations have demonstrated the distinct impacts of bile acid derivatives—metabolized by specialized gut bacteria, including *Clostridium scindens* and *Eubacterium* spp.—on the differentiation of Tregs (Yoon et al., 2023). Isoallolithocholic acid (isoalloLCA) enhances Tregs development by generating mitochondrial reactive oxygen species (ROS) (mitoROS), resulting in enhanced Forkhead box protein P3 (FOXP3) expression and Tregs differentiation regardless of the microbiome structure (Yoon et al., 2023). IsoalloLCA stimulates the nuclear hormone receptor Nr4a1 (nuclear receptor subfamily 4, group A, member 1) at the molecular level, leading to the creation of a permissive chromatin structure in the promoter region of the transcription factor FOXP3 (Li W. et al., 2021). Through the process of microbial epimerization, isodeoxycholic acid (isoDCA), along with isoalloLCA, reduces the immunostimulatory effects on DCs by acting on the FXR. IsoDCA induces extrathymic differentiation of RAR-related orphan receptor gamma (RORγt)+ Tregs cells by promoting Foxp3 induction and Tregs differentiation in a CNS1-dependent manner (Campbell et al., 2020). The 3-oxoLCA interacts directly with RORγt, a crucial transcription factor that promotes the development of Th17 cells, to hinder the process of Th17 cell differentiation. The degree of severity of the murine acute enteritis mouse model was improved with the treatment of 3-oxoLCA (Hang et al., 2019). These findings emphasize the regulatory potential of bile acid metabolites from specific commensal taxa capable of 7α-dehydroxylation and epimerization.

Tryptophan metabolites, primarily produced by *Lactobacillus, Bifidobacterium*, and *Clostridium* species, are crucial in the formation of ILC3 (Wang et al., 2023). AhR activation is necessary for the generation of IL-22 in ILC3s, which is facilitated by AhR ligands derived from the microbiota (Zelante et al., 2013; Gutiérrez-Vázquez and Quintana, 2018). Tryptophan metabolites have a role in the regulation of mucosal immunity by affecting the activity of the AhR. The diminished AhR function in AhR knockout mice was associated with a decrease in ILC3 and an exacerbation of inflammatory disorders (Lamas et al., 2016). Disrupting tryptophan metabolism in the gut microbiota leads to a decrease in IL-22 levels in the intestines. On the other hand, activating the AhR in type 3 ILC3 stimulates the production of IL-22. This process helps regulate the intestine's immunological response and protects the integrity of the intestinal barrier. AhR also significantly impacts the differentiation of ILC3s (Lee et al., 2011; Qiu et al., 2012). AhR ligands are necessary for the development of IL-22-producing ILC3s, particularly in the early period after birth (Gomez de Agüero et al., 2016). Functionally, AhR is involved in the formation of ILC3 through its interaction with Runx3 and RORγt. Additionally, AhR regulates the expression of Notch and c-Kit (Kiss et al., 2011; Qiu et al., 2012; Ebihara et al., 2015). Significantly, decreased AhR signaling may lead to changes in the cellular populations of ILC3 and ILC1. Furthermore, AhR may induce the production of IL-22 in Th17 cells (Veldhoen et al., 2008).

### 6.3 Monocytes

Bile acid metabolites play a crucial role in maintaining the tolerogenic phenotype of macrophages via the bile acid receptor TGR5 (GPBAR1) (Wang et al., 2023). TGR5 can suppress the production of cytokines by macrophages after exposure to LPS. The presence of TGR5 deficiency in mice might potentially enhance the inflammation caused by LPS in the liver, while the ability of a TGR5 agonist to reduce the levels of inflammatory mediators could be nullified (Wang et al., 2011). TGR5 can inhibit inflammation that is reliant on the NLRP3 inflammasome, as shown by studies (Pols et al., 2011; Guo et al., 2016b). Actually, the TGR5 ligands and SBAs DCA and LCA may naturally limit the activation of NLRP3 by promoting TGR5-cAMP-PKA-dependent ubiquitination of NLRP3 (Pols et al., 2011; Guo et al., 2016b). Increased amounts of cAMP inside the cells may trigger the phosphorylation and ubiquitination of NLRP3, preventing NLRP3-dependent inflammation and metabolic problems associated with NLRP3. Activation of TGR5 also stimulates the polarization of macrophages toward a tolerogenic phenotype, as shown in Macs (Shi et al., 2020). The order of hierarchy for TGR5 activation is as follows: LCA > deoxycholic acid (DCA) > chenodeoxycholic acid (CDCA) > ursodeoxycholic acid (UDCA)> cholic acid (CA) (Di Vincenzo et al., 2022). Furthermore, FXR plays a crucial role in preserving the tolegeronic phenotype of Macs, as shown by studies on FXR knockout mice (Vassileva et al., 2006; Fiorucci et al., 2018). Additionally, FXR serves as a significant inhibitory regulator of NLRP3 by interacting directly with NLRP3 and caspase-1 (Hao et al., 2017). The transcription factor FXR is able to stabilize nuclear receptor corepressor 1 (NCOR1) complexes on the promoters of iNOS (nitric oxide synthase) and IL-1β genes, which are attracted to the promoters of these genes (Vavassori et al., 2009). A number of genes that are associated with inflammation, including iNOS, TNFα, and IL-1β, are characterized by NCoR1 in their promoter regions. These promoter areas are connected to an NF-κB-responsive element. By activating SOCS3 (suppressor of cytokine signaling 3), CYP450 (cytochrome P450), and FGF19 (fibroblast growth factor 19), FXR can reduce inflammation. Additionally, SHP (Src homology-2 containing protein tyrosine phosphatase) can inhibit NF-κB, AP-1 (activator protein-1), and NLRP3 (Wang et al., 2023). There is also a nuclear receptor known as PXR that binds to LCA (Wang et al., 2023). There is a possibility that activation of PXR might reduce the expression of IL6, TNFα, and IL8 (Wang et al., 2023).

SCFAs, particularly butyrate, produced by commensal bacteria such as *Faecalibacterium prausnitzii*, also modulate monocyte/macrophage activity (Akhtar et al., 2022). In a study, Cox et al. (2009) discovered that having access to SCFAs in the body leads to anti-inflammatory effects via controlling the synthesis of cytokines and prostaglandin E2 (PGE2) by human monocytes. They found that the presence of these fatty acids led to a spike in the production of PGE2, indicating a synergistic effect when the fatty acids were incubated with LPS (Cox et al., 2009). When cells were exposed to SCFAs after being primed with LPS, there was a decrease in IL-10 production. Additionally, a dose-dependent relationship was established between the reduction of C-C motif chemokine ligand 2 (CCL2) production and the reported effects. There is a connection between each of these discoveries and the cytokine profile. The findings obtained from peripheral blood mononuclear cells were similar, with the added benefit of a reduction in the production of TNF-α and IFN-γ that was caused by LPS (Cox et al., 2009). On the other hand, different research indicated that SCFAs had a pro-inflammatory impact on human peripheral blood mononuclear cells. It was noted that this event occurred either when SCFAs were present independently or, in rare instances, when they were coupled with TLR agonists (Mirmonsef et al., 2012). However, it has also been demonstrated that butyrate may block the migration of RAW264.7 cells and rat thioglycolate-elicited macrophages triggered by LPS (Maa et al., 2010). The findings of research that was carried out by Chang et al. (2014), who conducted a study that demonstrated that butyrate has anti-inflammatory effects on bone marrow, indicated that butyrate has the ability to reduce inflammation in macrophages that are produced from bone marrow. To be more specific, they discovered that butyrate could inhibit the synthesis of nitric oxide (NO), IL-6, and IL-12p40 in response to LPS, whereas it had no impact on the production of TNF-α or the cytokine CCL2 (Chang et al., 2014). Propionate and acetate, unlike butyrate, did not demonstrate the same anti-inflammatory characteristics (Chang et al., 2014). The findings that were obtained from the *in vitro* incubation of macrophages that originated from the colonic lamina propria with butyrate and LPS were similar to the results that were seen after the oral administration of this SCFA (Chang et al., 2014). In their study, Chang et al. (2014) established that the mechanism of action of butyrate includes the inhibition of HDACs. In summary, microbiota-derived metabolites—including tryptophan catabolites, bile acids, and SCFAs—profoundly influence monocyte/macrophage biology through receptor-mediated, transcriptional, and epigenetic mechanisms.

### 6.4 Polymorphonuclear leukocytes (PMN)

Neutrophils, the most abundant PMNs, are critical first responders in host defense and are tightly regulated by microbial signals (Fernandez-Sanchez et al., 2024; Liu and Shi, 2025). Microbiota-derived SCFAs are responsible for regulating several neutrophil functions (Mei et al., 2012). They affect the recruitment of neutrophils by influencing the production of inflammatory chemicals, such as IL-17 and TNF-α (Sina et al., 2009). It has been found that the activation of the GPR43 receptor on neutrophils by SCFA is responsible for controlling neutrophil chemotaxis (Sina et al., 2009; Vinolo et al., 2011a). Furthermore, synergistic fatty acids have the potential to influence the actions of neutrophils, including their capacity for phagocytosis as well as their ability to generate and release ROS and NO (Rodrigues et al., 2016). However, it seems that the apoptosis caused by butyrate and propionate is not dependent on SCFA receptors. Instead, it is dependent on caspase activation rather than the Gαi/o and Gq pathways in both activated and non-activated neutrophils (Aoyama et al., 2010). The antibacterial effect of butyrate is thought to be caused by an increase in the recruitment of neutrophils (Ney et al., 2023). Furthermore, it was shown that SCFAs were specific in their ability to inhibit the production of NO by neutrophils (Vinolo et al., 2011b). Butyrate was shown to be effective in preventing the movement of neutrophils and the formation of neutrophil extracellular traps (NETs) (Li G. et al., 2021). These findings provide insight into the relevance of certain environmental cues that affect SCFA-related signaling pathways and the actions that are triggered by those pathways. SCFAs have been under increased examination in recent years (Fu et al., 2021) due to the potential therapeutic applications they offer in the treatment of a variety of ailments, particularly those associated with the dysfunction of neutrophils, notably inflammatory and autoimmune disorders. Both the efficiency of butyrate treatment in limiting the recruitment of myeloid and lymphoid cells into the colonic mucosa and the participation of immune cell penetration in an inflammatory response throughout the evolution of colitis have been thoroughly investigated in earlier research (Tsou et al., 2007).

Bile acids, specifically SBA LCA, can inhibit the release of IL-1β by the NLRP3 inflammasome, which ultimately results in a decrease in the recruitment of neutrophils during the inflammatory process (Guo et al., 2016a). Furthermore, mice that are lacking the FXR, which is the receptor that detects bile acids, have been shown to have enhanced osteopontin generation from Natural killer T (NKT) cells in the liver (Diao et al., 2004; Mencarelli et al., 2009). This, in turn, leads to an increase in neutrophil-mediated liver damage in a model of autoimmune hepatitis (Diao et al., 2004; Mencarelli et al., 2009). Throughout inflammatory processes, AhR ligands that are produced from the microbiota boost the immune-suppressive capabilities of Tregs, which in turn have an indirect effect on neutrophil recruitment and activation (Zelante et al., 2013; Ye et al., 2017). Even though it has been hypothesized that AhR ligands formed from the host might inhibit chemotaxis in neutrophils during inflammatory processes, it is yet unclear if microbiota-derived AhR ligands can directly connect with neutrophils (Loughman and Hunstad, 2012; Loughman et al., 2016). In summary, commensal-derived microbial metabolites—particularly SCFAs, bile acids, and tryptophan catabolites—act through well-defined receptors to modulate neutrophil activity, suppress inflammation, and fine-tune host defense mechanisms.

### 6.5 Dendritic cells

Both butyrate and propionate have been shown to inhibit the formation of DCs from murine bone marrow cells (Wilson et al., 2022). To be more specific, murine bone marrow cells that have been cultivated in the presence of granulocyte-macrophage GM-CSF and either butyrate or propionate have a reduced ability to differentiate into CD11c+ cells when compared to cells that have been cultured in the absence of SCFAs (Singh et al., 2010). It has been discovered that similar outcomes occur during the process of differentiation of monocyte-derived DCs (moDCs) from human monocytes (Millard et al., 2002; Säemann et al., 2002). This particular phenotype is dependent on SLC5A8 and GPR109A, and it has been discovered that cells lacking either of these receptors exhibit enhanced Bone marrow-derived DC (BMDC) differentiation, although it is not completely recovered (Singh et al., 2010). However, more comprehensive genome-wide transcription and chromatin accessibility studies are needed to fully comprehend the mechanisms at play (Singh et al., 2010). It is believed that butyrate and propionate hinder *in vitro* DC differentiation by controlling transcription factors essential for the development of DCs. This is accomplished through the inhibition of HDAC. For the formation of DC, the research made use of GM-CSF rather than Flt3 ligand (Flt3L), which is an important point to note. DCs that are formed in these cultures are unique from one another in terms of their development and phenotype, with the former aligning more closely to inflammatory DCs and the latter aligning more closely to steady-state DCs (Naik et al., 2005; Xu et al., 2007). There is also a lack of clarity about whether or not SCFA administration affects the formation of Flt3L-dependent DCs *in vitro*. This is a significant issue in relation to diets that are rich in fiber and the effect that they have on the immune system (Trompette et al., 2018). SCFAs, in contrast to circumstances that are steady-state, encourage the production of DC bone marrow progenitor cells *in vivo* when inflammatory conditions are present. Propionate treatment of mice that were exposed to house dust mites increased the number of common DC progenitors (CDPs) and macrophage-DC progenitors (MDPs), which in turn promoted the formation of moDCs (Trompette et al., 2014). When mice are given a diet that is rich in fiber, it has also been reported that the number of MDPs increases (Trompette et al., 2018). Influenza infection, on the other hand, leads to an increase in the production of Ly6C-monocytes, which are responsible for developing alternatively activated macrophages rather than DCs. Interestingly, both findings reveal a dependence on the expression of GPR41, even though the result of microbiota-altered hematopoiesis is greatly reliant on the setting.

Several mechanisms may be responsible for the effects of SCFA on DC activation. Firstly, the activation of moDCs in the presence of SCFA (e.g., butyrate) leads to reduced extracellular acidification rate (ECAR) and oxygen consumption rate (OCR) (Wilson et al., 2022). This behavior is comparable to that of moDCs that have been activated in the presence of glycolytic inhibitors, which raises the potential that butyrate may control glycolysis. The metabolic alterations that are caused by butyrate have also been established in colonocytes and T cells; however, the effects of butyrate on DCs have not yet been investigated (Donohoe et al., 2011; Trompette et al., 2018; Bachem et al., 2019). GPRs are the means via which SCFAs may affect DCs. However, it has been shown that butyrate has the ability to inhibit the translocation of NF-κB to the nucleus in murine colonocytes and BMDCs via the GPR109A pathway. In the presence of butyrate, DCs that were stimulated with LPS would exhibit a decreased production of proinflammatory cytokines, which might be attributed to the impairment of NF-κB activation (Säemann et al., 2002; Thangaraju et al., 2009). Thirdly, it is well-established that SCFAs block HDACs, which allows them to control the acetylation of histones as well as the acetylation of transcription factors. There is an increase in the amount of histone acetylation in moDCs that have been incubated in the presence of butyrate and, to a lesser degree, propionate (Kaisar et al., 2017). Impairment of DC activation, which is defined as a reduction in the upregulation of costimulatory molecules, is found when DCs are treated with HDAC inhibitors in a manner that is comparable to the findings reported when DCs are treated with butyrate (Kaisar et al., 2017). Based on this, it seems that the suppression of HDAC that is produced by butyrate may also contribute to decreased DC activation. The findings indicate that the microbial metabolites of healthy individuals have a tendency to reduce the amount of classical DC activation and also show a clear need for future research into precisely how the metabolites interact with and influence DCs, with a focus on the impacts on DC transcriptional, signaling, and metabolic profiles.

### 6.6 Platelets

An intriguing junction between the domains of microbiology and hematology is represented by the microbiota metabolites and the influence that they have on platelets and thrombocytosis (Yoon et al., 2019). The findings indicate that metabolites produced from microbiota have the potential to alter platelet function via a variety of methods (Yoon et al., 2019). When seen from this perspective, the previously documented trimethylamine N-oxide (TMAO) produced by commensal and opportunistic bacteria from dietary choline and L-carnitine-mediated increase in platelet responsiveness is believed to be generated by an increased release of calcium (Ca2+) from intracellular reservoirs (Zhu et al., 2016). This, in turn, promotes platelet aggregation, which in turn leads to the development of a thrombus (Zhu et al., 2016). The impact of TMAO on platelet activity was investigated using robust liquid chromatography-mass spectrometry (LC-MS)/MS analysis, which accounted for both *in vivo* and *in vitro* conditions while minimizing pre-analytical impacts (Emonds et al., 2022). Emonds et al. (2022) investigated the influence of TMAO on platelet activation through a structured series of experiments. In their first experimental series, ristocetin stimulation induced a consistent rise in TXB2 levels, with only minor day-to-day variation in a single individual. The second series expanded the analysis to multiple participants and revealed greater inter-individual variability; notably, participants with high endogenous TMAO concentrations exhibited a stronger TXB2 response compared with those with lower levels (Emonds et al., 2022). These observations prompted a third series, which directly assessed the relationship between plasma TMAO and TXB2 production. While the second series suggested a potential correlation between TMAO and ΔTXB2, this association may have been influenced by the limited sample size or unmeasured variables. Nonetheless, the sustained presence of TMAO in circulation could exert a stimulatory effect on platelets that short-term experimental exposure may not capture (Emonds et al., 2022). Nevertheless, the prolonged presence of TMAO in circulation may exert a stimulatory effect on platelets that cannot be replicated by short-term experimental exposure.

Thrombus development often involves a series of signaling channels that enhance the process (Rivera et al., 2009). Koltai et al. (2017) replicated this process using ristocetin or adenosine diphosphate (ADP). During this procedure, the arachidonic acid metabolites undergo conversion by several enzymes, such as COX, LOX, and CYP, either in the blood or in blood cells (Koltai et al., 2017). In platelet metabolism, numerous eicosanoids, along with thromboxanes, are present and contribute to platelet activation, blood clot development, and atherothrombosis advancement (Badimon et al., 2021; Zhou et al., 2021). Emonds et al. (2022) observed notable elevations in the levels of compounds derived from the LOX and COX pathways, whereas the levels of compounds produced by CYP metabolism and early precursors were mostly reduced. These effects seem to be associated with enzymes but may not necessarily be influenced by the precursor fatty acid. Nevertheless, the conflicting patterns they exhibit prevent a definitive explanation. 12-HHT is a high-affinity ligand for BLT2 receptors and is generated in significant amounts by activated platelets (Saeki and Yokomizo, 2017). The elevated synthesis of 12-HHT and 12-HETE in human platelets indicates a higher abundance of unbound arachidonic acid, as previously seen by the activation of GPR91 (Tang et al., 2020). Prior studies have previously revealed alterations in COX-products, including TXB2 and 12-HHT, as well as LOX-derived metabolites during activation of platelets, which supports our research results (Rund et al., 2020). This statement provides more evidence to support the argument that stimulating biological responses via platelet activation might uncover possible consequences of thrombo-inflammation (Cebo et al., 2020). DHETs have been identified as substances that protect against the natural regulation of blood flow in the small blood capillaries of the heart, which might explain why their levels were lower in the tests reported (Larsen et al., 2006). Given the lack of a circadian rhythm, the absence of any direct impact from a vegetarian diet, and the smaller fluctuation in TMAO plasma levels within individuals compared to across individuals during the day, it has been confidently stated that a blood sample for measuring TMAO may be taken at any time without considering the time of day. The precise functions of specific metabolites in the metabolic pathways being addressed have yet to be determined, unlike the well-established role of TMAO.

In addition, research demonstrated that propionate elevated the expression of CD61 on the cell surface, which is a recognized indicator of megakaryocyte development (Dürholz et al., 2022). Therefore, it seems that propionate impacts the megakaryocyte maturation process in a laboratory setting. However, when seen in living organisms, Dürholz et al. (2022) found a decrease in the occurrence of megakaryocytes and their precursor cells. The reduced presence of megakaryocytes in living organisms, as shown in mice lacking the kit gene and resistant to arthritis, may partially account for the decreased severity of arthritis symptoms during propionate therapy. This might be attributed to a potential decrease in the activation of synovial fibroblasts reliant on IL-1. Nevertheless, Dürholz et al. (2022) failed to show evidence establishing a direct correlation between the impact of propionate on megakaryocytes and their progenitors and the symptoms of arthritis. Hence, it would be intriguing to investigate the impact of propionate on the whole process of differentiation from HSCs to megakaryocytes, using HSC tracing as previously outlined by Grinenko et al. (2018). In summary, microbiota metabolites play a complex and multifaceted role in regulating platelet function. By modulating inflammatory pathways, oxidative stress, and directly interacting with platelet receptors, these metabolites can influence platelet activation, aggregation, and overall cardiovascular health.

## 7 Context-dependent effects of microbial metabolites on hematopoiesis and immunity

An important consideration in interpreting microbiota-derived metabolites is that their biological effects are not necessarily fixed or universally beneficial or detrimental. Rather, metabolite activity is determined by a combination of concentration, chemical identity, cellular target, receptor expression, disease stage, microbial source, and host biological background (Mester et al., 2026). Thus, the same metabolite can produce distinct—and occasionally opposing—responses depending on the cellular and inflammatory environment in which it acts (Burrichter et al., 2025). This context dependency is particularly evident for SCFAs. Although acetate, propionate, and butyrate are widely recognized for promoting immune tolerance and regulatory T-cell differentiation, their effects vary according to the specific SCFA, exposure level, receptor availability, and responding cell type (González-González et al., 2026). For example, acetate and propionate can promote expansion of pre-existing colonic Tregs, whereas propionate and butyrate can preferentially support *de novo* differentiation of naïve T cells into Tregs, demonstrating that even closely related SCFAs can engage distinct biological programs (Arpaia et al., 2013; Meyer et al., 2020). In contrast, studies using human peripheral blood mononuclear cells have reported that SCFAs can, under certain experimental conditions, exert pro-inflammatory effects, whereas butyrate suppresses inflammatory mediator production in bone-marrow-derived macrophages (Cox et al., 2009; Ji et al., 2016; Saleri et al., 2022). The cellular target and receptor repertoire represent another major determinant of metabolite activity. SCFAs can signal through FFAR2/GPR43 and other G protein-coupled receptors while also exerting receptor-independent epigenetic effects through HDAC inhibition (Martin-Gallausiaux et al., 2021). Consequently, cells expressing different combinations or levels of these receptors may respond differently to an identical metabolite concentration. This principle is particularly evident in dendritic-cell biology, where butyrate and propionate can inhibit dendritic-cell differentiation *in vitro*, whereas propionate can increase dendritic-cell progenitors under inflammatory conditions *in vivo*. The distinction between GM-CSF-dependent inflammatory DCs and Flt3L-dependent steady-state DCs further illustrates how cellular developmental context can determine the response to the same metabolite (Wilson et al., 2022; Andrusaite et al., 2024). Metabolite concentration and systemic availability also influence biological outcome (Azcona-Granada et al., 2025). Local concentrations within the intestinal lumen may differ substantially from those reaching the circulation and bone marrow, while dietary, microbial, metabolic, and disease-associated changes can markedly alter metabolite availability (Wang et al., 2026a). Therefore, observations obtained using pharmacological concentrations *in vitro* should not automatically be extrapolated to physiological exposure *in vivo*. Indeed, the manuscript indicates that dietary interventions can markedly increase SCFA availability, emphasizing that the magnitude of exposure is an important component of metabolite-mediated signaling.

Disease stage and inflammatory status provide an additional layer of regulation. During homeostasis, microbial metabolites may support immune tolerance, tissue integrity, and balanced hematopoiesis, whereas during infection, transplantation, or chronic inflammation, the same signaling pathways may participate in emergency hematopoiesis or alter lineage commitment (Kim and Kamada, 2023). For example, SCFAs can suppress excessive inflammatory responses but may also enhance the production of dendritic-cell progenitors during inflammatory challenge (Liu et al., 2023). Similarly, microbial signals that are required for appropriate emergency myelopoiesis during infection may become pathogenic when excessive or chronically activated. These observations indicate that metabolite effects should be interpreted in relation to the pre-existing inflammatory state rather than considered intrinsically anti- or pro-inflammatory. The microbial source and metabolic context are also critical (Chilloux et al., 2016). A metabolite should not be considered independently of the microbial community that produces it, because different taxa possess distinct metabolic capacities and generate different concentrations, combinations, and ratios of metabolites. The findings therefore distinguish commensal-derived metabolites from products associated with pathobionts or dysbiotic communities. This distinction is particularly evident in hematopoiesis: commensal-derived SCFAs can participate in homeostatic regulation, whereas LPS derived from facultative pathobionts can suppress bone-marrow erythropoiesis through TLR4/MyD88 signaling in erythroblastic-island macrophages (Krishnamurthy et al., 2023). Similarly, dysbiosis may simultaneously decrease protective metabolites and increase inflammatory microbial products, thereby changing the overall signaling balance rather than simply producing a deficiency of a single beneficial metabolite. Finally, host genetic and biological background may influence metabolite responsiveness by determining receptor expression, intracellular signaling competence, metabolic capacity, and the basal state of hematopoietic and stromal cells (Spivak et al., 2022; Zhou et al., 2026). This is particularly relevant for metabolites that act through genetically regulated sensing pathways such as AhR, FXR, TGR5, FFARs, and TLRs. The functional importance of host sensing is illustrated by the observation that loss of AhR activity is associated with HSC exhaustion and dysregulated hematopoiesis, whereas TGR5 deficiency can enhance inflammatory responses to LPS (Sun et al., 2026). Thus, inter-individual differences in host sensing pathways may contribute to variability in responses to the same microbial metabolite. Collectively, these observations support a context-dependent model of microbiota–metabolite signaling, in which the biological outcome is determined by the interaction among metabolite identity and concentration, microbial origin, receptor and cellular target, host genetic and metabolic background, disease stage, and the pre-existing state of the hematopoietic niche. Accordingly, microbial metabolites should not be classified simply as “beneficial” or “harmful.” Instead, their effects should be interpreted as conditional outputs of a dynamic host–microbiota system. This framework provides a more mechanistic explanation for why alterations in the same metabolite pathway may produce different phenotypes across hematological disorders and why therapeutic manipulation of microbial metabolites will likely require disease-, dose-, cell-, and patient-specific strategies.

## 8 Disease-specific mechanisms linking microbial metabolites to hematological-associated pathogenesis

The contribution of gut microbiota-derived metabolites to hematological disease is best understood as a context-dependent interaction between microbial metabolic output, host sensing pathways, the bone marrow microenvironment, and the intrinsic state of hematopoietic cells. Although dysbiosis and altered metabolite profiles are observed across several hematological disorders, the downstream consequences differ according to the metabolite involved, its concentration and systemic availability, the responding cell type, receptor expression, and the inflammatory or malignant state of the hematopoietic niche (Valaei et al., 2026). Microbial metabolites can therefore influence disease through either direct regulation of hematopoietic cells or indirect remodeling of the cellular and inflammatory environment in which hematopoiesis occurs. Taken together, these disease models demonstrate that the same microbiota–metabolite axis can produce distinct pathological outcomes. In anemia, inflammatory microbial products suppress erythropoiesis through niche inflammation, whereas SCFAs can exert more direct regulatory effects on erythroid precursors (Coll et al., 2024). In GVHD, reduced butyrate availability and increased TMAO can contribute to pathology through different mechanisms involving epithelial integrity and macrophage–T-cell inflammatory circuits (Hayase and Jenq, 2020; Hong et al., 2021). In hematological malignancies, microbial metabolites may influence malignant-cell survival, immune surveillance, inflammatory signaling, and treatment response (Guevara-Ramírez et al., 2024). Thus, disease phenotype is determined not simply by whether a metabolite is increased or decreased, but by the interaction between metabolite identity, receptor/signaling pathway, cellular target, hematopoietic lineage, and the pre-existing state of the bone marrow niche. In recent years, significant focus has been given to the relationship between the human microbiota and blood cells, which are regulated by the generation of metabolites in different disorders (Man et al., 2020; Afzaal et al., 2022). This section will examine the influence of microbial metabolites on blood cells in different disorders (Table 2).

**Table 2:** Function of microbiota-derived metabolites on hematopoiesis in diseases.

Disease	Study setting	Metabolite	Function	Outcome	References
Colitis	*In vivo*	Propionate	Enhancing intestinal barrier function, reducing inflammation, and alleviating oxidative stress	Sodium propionate mostly helps colitis by improving the function of the intestinal barrier, lowering inflammation, and reducing oxidative stress. These effects were mainly caused by the STAT3 signaling system.	Tong et al., 2016
Colitis	*In vivo*	Butyrate	Enhanced mucosal barrier function, reduced permeability, mitigated neutrophil infiltration	Butyrate increased the number of living colonocytes, improved the function of the mucosal barrier, decreased permeability, stopped neutrophils from entering, and changed the reactions of heat shock proteins and NF-κB activation in DSS-induced colitis.	Venkatraman et al., 2003
Ulcerative colitis (UC)	Clinical, *in vitro*	Butyrate	Anti-inflammatory effects	Since the main cause of inflammation in UC is cytokines produced by macrophages, the anti-inflammatory effects of butyrate may be due in part to its capacity to block the activation of NF-κB in these macrophages.	Lührs et al., 2002
Inflammatory bowel disease (IBD)	*In vitro*	Butyrate	Neutrophil activity	Butyrate has been found to have significant potential in affecting neutrophil activity and reducing tissue damage associated with the excessive production of reactive oxygen species (ROS) by neutrophils in the context of colitis.	Sacco et al., 2017
Rheumatoid arthritis	Clinical and *in vivo*	Butyrate	Histone deacetylase (HDAC), regulatory T cells (Tregs), and Follicular helper T (TFH)	Administering butyrate as a dietary supplement in a mouse model provided anti-inflammatory benefits by restoring the balance of TFH cells and regulatory Tregs, while also reducing antibody production.	He et al., 2022
Collagen-induced inflammatory arthritis model (CIA)	*In vivo*	Propionate	Megakaryopoiesis	The ingestion of propionate led to a considerable decrease in the frequency of the megakaryocyte-erythrocyte progenitor (MEP) population in the bone marrow of both CIA and naïve mice.	Dürholz et al., 2022
Inherited hemoglobin disease	*In vivo*	Butyrate	Fetal hemoglobin induction	Sodium butyrate up-regulated the gamma chain gene and changed miRNA expression.	Tayebi et al., 2017
Cooley's anemia	*In vivo*	Butyrate	Induction of *γ-globin*	Novel binding of transcription factors, such as αCP2, which activates *γ-globin*, was observed only in the butyrate-responsive patients throughout therapy. These binding events were located in specific regulatory areas of the γ- and *β-globin* gene promoters.	Ikuta et al., 1998
Congenital hemolytic anemia	*In vitro*	Butyrate	Hemoglobin chain synthesis	Butyrate has the potential to regulate the synthesis of hemoglobin chains after the process of transcription. This process may be influenced by the stability of the transferrin receptor and globin RNA.	Reinhardt et al., 2001
β- Thalassemia	*In vitro*	Butyrate and valproic acid	Splicing modulation	The findings underscored the therapeutic promise of splicing regulation in treating genetic disorders resulting from splicing abnormalities.	Mahmoud et al., 2016
Renal anemia	Clinical Trial	SCFAs	-	The presence of dietary fiber in the diet positively impacted renal anemia in patients with End-stage renal disease (ESRD) by effectively controlling the composition of the gut microbiota and promoting the production of SCFAs.	
Melanoma	*In vitro* and *in vivo*	Butyrate	Tumor-associated macrophage (TAM) differentiation	Butyrate inhibited the growth of B16 melanoma by impeding the proliferation of TAM and decreasing the production of proteins that promote tumor growth in macrophages.	Xiong et al., 2015
Liver cancer	*In vivo*	T-w-MCA, taurodeoxycholic acid (TDCA), w-MCA, taurolithocholic acid (TLCA), and tauroursodeoxycholic acid (TUDCA)	hepatic CXCR6+ natural killer T (NKT) cells and IFN-γ	The increase of NKT cells was controlled by the production of CXCL16 in liver sinusoidal endothelial cells, which was regulated by the conversion of primary-to-secondary bile acids mediated by the gut microbiome.	Ma et al., 2018
Acute pancreatitis	*In vivo*	Acetate	Inflammation	Neutrophils have a crucial impact on the progression of acute pancreatitis. The decrease in neutrophils achieved by administering Parabacteroides and acetate strongly correlated with the reduction of pancreatic inflammation in heparanase-transgenic animals.	Lei et al., 2021
Multiple sclerosis	Clinical and *in vitro*	Acetate and butyrate	IL−10–producing B-cells, class-switched memory B-cells, and polyclonally-activated B-cells	Butyrate levels are positively correlated with IL-10-producing B-cells and negatively correlated with class-switched memory B-cells. The negative connection between acetate levels and polyclonally activated B-cell TNF production was observed.	Trend et al., 2021
Graft-vs.-host disease (GvHD)	*In vivo*	Chenodeoxycholic acid (CDCA), UDCA, TUDCA	Enhancing viability during exposure to pro-inflammatory cytokines and reducing antigen presentation in the intestine with a consequent decrease in apoptosis via FXR and TGR5	Allo-HCT causes a reduction in the amount of bile acids present in the body. The exogenous administration of bile acids, specifically TUDCA, decreases the occurrence of acute GvHD.	Haring et al., 2021
Allergic inflammation	*In vivo*	SCFAs	Generation of macrophage and dendritic cell (DC) precursors via GPR41	Mice treated with the SCFA propionate experienced changes in bone marrow hematopoiesis, including increased production of macrophage and DC precursors. These DCs seeded the lungs with high phagocytic capacity but lacked the ability to promote T helper type 2 cell effector function.	Trompette et al., 2014

### 8.1 Anemia

Li et al. (2022) demonstrated that adequate dietary fiber consumption enhanced hemoglobin levels in individuals with anemia. This discovery is comparable to the outcomes documented in several prior animal and clinical investigations (Rossi et al., 2016; Carvalho et al., 2017; Paganini et al., 2017). Furthermore, the findings demonstrated that the intake of dietary fiber significantly increased hemoglobin levels in individuals with anemia, with an average rise exceeding 20% (Li et al., 2022). Moreover, Li et al. (2022) discovered that oral dietary fiber supplementation resulted in elevated levels of SCFA, particularly butyrate, in individuals with renal anemia. These results indicate that the rise in hemoglobin levels observed in patients with renal anemia who consume dietary fiber may be linked to an increase in butyrate and bacteria that are involved in its production (Duan et al., 2022; Ng et al., 2022). Butyrate is an extensively researched SCFA that has been shown to have various effects on disorders associated with anemia. Research conducted by Reid et al. (2014) discovered that derivatives of butyrate improved the symptoms of sickle cell anemia. Weinberg et al. (2005) showed that butyrate can enhance the production of γ-globin, hence improving thalassemia. Nevertheless, because of the multifactorial nature of renal anemia, it remains uncertain whether butyrate may effectively ameliorate the condition. Li et al. (2022) demonstrated the first evidence that butyrate, generated by gut bacteria's fermentation of dietary fiber, might ameliorate renal anemia in a clinical experiment. However, Li et al. (2022) discovered a reduction in the prevalence of *Blautia*, a bacterium that produces butyrate, in the dietary fiber group. However, the quantity of butyrate in the serum was shown to be increasing. Li et al. (2022) hypothesized that this might be linked to the capacity of *Bifidobacterium adolescentis, Lactobacillus*, and *Lactobacillaceae* to augment the generation of butyrate by other bacteria that produce butyrate.

In the process of inducing the β*-globin* gene, sodium butyrate has been deemed to be a low-risk and highly effective substance (Weinberg et al., 2005). Tayebi et al. (2017) conducted research in which they found that the sodium butyrate group significantly increased the expression of g-globin. In contrast to the control group, sodium butyrate enhanced g-gene expression in CD133+ individuals in the sodium butyrate group (Panja and Basu, 2015). Research has shown that sodium butyrate is beneficial in treating individuals with sickle cell disease, including those who are resistant to hydroxyurea (HU). The discovery that sodium butyrate is an inducer of hemoglobin F was made by Tayebi et al. (2017). On the other hand, because of the negative effects of hemoglobin F inducers, it is necessary to discover an alternative to these products; microRNAs (miRNAs) have the potential to be a viable surrogate. It was recently found by Tayebi et al. (2017) that some microRNAs were up-regulated when sodium butyrate was present. As a result, these miRNAs have the potential to be used to promote β*-globin* gene expression in individuals suffering from β-thalassemia and sickle cell anemia.

### 8.2 Graft-vs.-host disease

Dietary habits influence the composition and diversity of the gut microbiota, which in turn modulates the production of microbial metabolites—an emerging factor in the pathogenesis and severity of GVHD (Hazenberg, 2018; Shono and van den Brink, 2018; Godefroy et al., 2024). Metabolites such as SCFAs and indole derivatives have been shown to alleviate GVHD by preserving intestinal epithelial integrity and modulating immune pathways, including type I IFN signaling (Mathewson et al., 2016; Swimm et al., 2018). Wu et al. (2020) demonstrated that TMAO exacerbated GVHD pathology in mice. Interestingly, 3,3-dimethyl-1-butanol (DMB), a structural analog of choline, counteracted this effect, highlighting the therapeutic potential of targeting specific microbial metabolic pathways. The exacerbation of GVHD by TMAO was not directly due to enhanced T cell activation but rather associated with macrophage-driven M1 polarization, leading to increased Th1 and Th17 responses (Wu et al., 2020). Regarding the antigen-presenting necessity for T-cell-mediated GVHD, Wu et al. (2020) concentrated their attention on the macrophage activation function in TMAO-enhanced GVHD severity.

It was shown that the percentages of M1 macrophages were elevated in GVHD mice that were given TMAO. Additionally, the signature of polarized M1 cytokines that are produced from BMDMs was shown to be enhanced after treatment with TMAO. The results suggest that TMAO might be responsible for controlling the polarization of M1 macrophages, which in turn regulates the response of Th1 and Th17 activation (Wu et al., 2020). In the work conducted by Wu et al. (2020) it was shown that TMAO can trigger the translocation of NF-κB and activate the NLRP3 inflammasome. The deletion of this inflammasome in BMDMs resulted in the obstruction of TMAO-induced M1 polarization. A recent discovery made in endothelial cells showed that TMAO can activate NLRP3 inflammasomes via the generation of endogenous mitochondrial ROS (Chen et al., 2017; Ke et al., 2018). The activating signal, which is triggered by adenosine triphosphate, calcium influx, and the formation of reactive oxygen species, leads to the cleavage of caspase-1 and the release of IL-1β, which is a cytokine that generates proinflammatory responses (Lang et al., 2018). There was a correlation between the treatment of macrophages with TMAO and an increase in mitochondria-derived ROS, which was related to a reduction in mitochondrial membrane potential and an impairment in mitochondrial respiration. Wu et al. (2020) reported that TMAO promoted the activation of aortic macrophages and elevated the expression of the IL-1β gene in atherosclerotic plaque (Fatkhullina et al., 2018). Surprisingly, TMAO reduced survival rates in WT mice but not in *Nlrp3–/–* animals. Wu et al. (2020) revealed that choline-generated TMAO, as a stimulator in GVHD development, promotes M1 macrophage polarization, activating Th1 and Th17 responses. Their work fills in the gaps between the host choline diet, microbial metabolites, and GVHD severity, demonstrating the possibility of treating GVHD by regulating choline consumption. Blocking the TMAO pathway in conjunction with other techniques, such as SCFA protection, would aid in the orchestration of microecological balance, potentially serving as a targeted avenue to reduce GVHD and other inflammatory disorders.

Recent clinical studies have already linked reduced gut microbial diversity and butyrate-producing bacteria to poor outcomes post-allo-HCT (Holler et al., 2014; Weber et al., 2016). A recent study by Mathewson et al. (2016) explored the role of microbial metabolites in acute GVHD severity. Their findings focused on SCFAs and long-chain fatty acids (LCFAs) from the skin, gut, and liver after experimental allo-HCT (Mathewson et al., 2016). The research found that of all the SCFAs and LCFAs from different tissues and feces, only SCFA butyrate was substantially reduced in the IECs. The decrease in IEC butyrate was linked to a decrease in diversity in microbial communities and in the family of bacteria that make butyrate from resistant starch. Butyrate treatment significantly raised the expression of its surface receptor, GPR43, and transporter protein, SLC5A8 (Mathewson et al., 2016). Most importantly, preserving butyrate levels, either through direct butyrate administration or by altering the microbiome to include more butyrogenic bacteria, such as the *Clostridia* strains, minimized intestinal barrier destruction, acute GVHD-specific destruction, and increased survival following allo-HCT. Butyrate boosted histone acetylation and was quickly degraded by IECs during allo-HCT (Mathewson et al., 2016). *Ex vivo* investigations revealed that treating IECs with SCFA butyrate reduced apoptosis when grown with alloreactive T cells (Mathewson et al., 2016). Surprisingly, acute GVHD decreased even after the donor Tregs were removed from the donor cells. These data collectively suggest that while mouse models offer valuable mechanistic insights, the translation to clinical application requires consideration of interspecies metabolic differences and validation in human systems. Future studies should integrate dietary modulation, microbial engineering, and personalized microbiome–metabolite profiling to develop targeted interventions that minimize GVHD in human patients undergoing allo-HCT.

### 8.3 Hematologic malignancies and solid cancers

Emerging research underscores the critical role of the gut microbiota and its metabolites in modulating the development and progression of hematologic malignancies, particularly leukemia and lymphoma (Uribe-Herranz et al., 2021; Xu et al., 2023; Liu et al., 2025). Microbial metabolites, such as SCFAs, also contribute to hematologic tumor biology (Rodríguez-García et al., 2024; Konishi et al., 2025). *Clostridium leptum* maintains glucose levels in the gut microenvironment by generating butyrate through the pyruvate and acetyl-coenzyme A route (Linares and Hermouet, 2022). Butyrate, in turn, augments Treg numbers and inhibits IL-17 expression (Linares and Hermouet, 2022). In line with this, Calcinotto et al. (2018) showed that IL-17 deficiency in multiple myeloma (MM) mouse models—or antibiotic or anti-IL-17/IL-17R antibody treatment—retards MM development. Their work found *Prevotella heparinolytica* to be a primary microbial IL-17 producer stimulator (Calcinotto et al., 2018). Subsequently, butyrate-producing bacterial taxonomic composition in the gut microbiota of MM patients was linked with increased MRD negativity rates (Brevi et al., 2022). Pianko et al. (2019) further examined MM patients' gut microbiota composition and identified that MRD-negative responses were associated with higher ratios of *Eubacterium hallii* and *Faecalibacterium prausnitzii*. While *F. prausnitzii* secretes butyrate, *E. hallii* secretes propionate, both of which have immunomodulatory action, assisting in anti-inflammatory action (Pianko et al., 2019). Collectively, these findings emphasize the intricate relationship between the gut microbiome and hematologic malignancies.

Certain bacteria have been demonstrated to create tumor-suppressing metabolites such as SCFAs, while others produce tumor-stimulating metabolites such as SBAs (Mirzaei et al., 2021a). Substantial epidemiological research based on the relevance of SCFAs has linked a greater frequency of cancer to individuals with low-SCFA diets or a shortage in fecal SCFAs, particularly for breast and stomach cancers (Mirzaei et al., 2021a). Furthermore, research has revealed that fiber-rich diets are related to a decreased risk of acquiring cancer when compared to diets high in red meat consumption, which has been proven to raise the chance of developing cancer (Park et al., 2016; Shaw et al., 2017; Kopp et al., 2018). SCFAs impact both inside and outside cells because they attach to their receptors and function as epigenetic modulators (Kasubuchi et al., 2015). GPR109A forms Tregs and synthesizes both IL-10 and pro-inflammatory IL-18 (Thangaraju et al., 2009). These responses have been connected to cancer-causing occurrences, as demonstrated in *Niacr1*^−/−^ mice (Singh N. et al., 2014).

Pulliam et al. (2016) demonstrated that U937 and HL-60 cells create substantial amounts of CCL2 and CCL5 constitutively, stimulating monocyte chemotaxis. They also found that butyrate may reduce the concentrations of these chemokines that impact monocyte movement. As a result, butyrate has the capacity to alter the tumor microenvironment (TME), affecting monocyte recruitment to the tumor and perhaps reducing the number of tumor-associated macrophages (TAMs). When mediators of inflammation, such as cytokines, chemokines, and free radicals, remain in the body for an extended period of time, they induce oncogene stimulation, cancer cell proliferation, blood vessel development, and mutagenesis (Nakkarach et al., 2021).

In a study, Han et al. (2020) discovered that antibiotic therapy reduces the frequency of γδT and NK cells in the spleen while boosting both the number and frequency of IEL γδT cells. The largest increased populations are Vγ1 and Vγ4, rather than the most dispersed γδ subgroup in the small intestinal tract of mice (Vγ1-Vγ4-). The substance that is derived from mice that have been administered antibiotics can enhance the cytotoxicity of γδT cells (Han et al., 2020). Using gas chromatography (GC)-MS for metabolic profiling, Han et al. (2020) discovered that indoles are the most significant substances among tumor-bearing mice that were treated with antibiotics and those that were not treated with antibiotics when they were given γδT cell therapy. Furthermore, Han et al. (2020) discovered that 3-indolepropionic acid (IPA) is the molecule with the highest concentration in mice that were treated with antibiotics when combined with γδT cell therapy. The production of IPA, a significant metabolite, occurs in this manner. It has been shown that IPA plays a crucial role in safeguarding primary neurons and neuroblastoma cells from the oxidative damage that is triggered by β-amyloid protein and H2O2 (Chyan et al., 1999). In addition, the presence of high doses of IPA results in a rise in membrane fluidity, which is significant for purposes of cancer prevention (Karbownik et al., 2001). The origin of IPA remains indistinct, and it has been reported that IPA is abundant in germ-free mice as well as mice treated with broad-spectrum antibiotics, which suggests that the normal composition of the gut microbiota in mice may be disadvantageous to the generation of IPA and that when the quantity of bacteria is decreased by antibiotics, it can be produced more. These findings reinforce that microbial metabolites—whether SCFAs, SBAs, or indole derivatives—are not restricted in their influence to one cancer type; instead, they represent shared biochemical levers that the microbiota pulls to shape immunity in both hematologic and solid malignancies.

### 8.4 Inflammatory diseases

The gut microbiota and its metabolites exert a profound influence on hematologic cells, shaping their development, activation, and functional polarization during inflammatory diseases (Li G. et al., 2021; Wang et al., 2023). Studies have shown a correlation between the advancement of IBD and alterations in the patterns and composition of microbial metabolites (Rodríguez et al., 2020). Li G. et al. (2021) conducted research indicating that butyrate has a vital role in regulating neutrophil activity in patients with IBD. They found that butyrate significantly reduced the production of proinflammatory chemokines and cytokines by neutrophils (Li G. et al., 2021). Furthermore, Li G. et al. (2021) found that butyrate treatment of neutrophils resulted in the production of limited quantities of S100A8, S100A9, ROS, and MPO. Further work using Trichostatin A (TSA) and PTX revealed that butyrate inhibited the production of inflammatory mediators by neutrophils in a manner reliant on HDACi. In summary, the study conducted by Li G. et al. (2021) demonstrates that butyrate decreases the proinflammatory capacity of neutrophils, thereby lowering inflammation in the mucosal lining of individuals with IBD. These findings suggest that butyrate might potentially be utilized as a supplementary medication during the acute phase of intestinal inflammation to prevent further immune responses, since epithelial development and repair may be hindered during the chronic phase of inflammation.

Additionally, SCFAs were found to stimulate bone marrow hematopoiesis and reduce the capacity of DCs to initiate Th2 cell-mediated allergic airway inflammation (AAI) (Barr et al., 2018). The properties of butyrate, acetate, and propionate were first examined in neutrophils taken from human blood, since leukocytes are believed to be the primary cells responsible for the effects of IBD (Niederman et al., 1997). Tedelind et al. (2007) found that the chemicals butyrate, propionate, and acetate, in that order of potency, inhibit TNFα-mediated activation of the NF-κB pathway in a human colon cancer cell line. Finally, in an *in vitro* model of mouse experimental colitis, acetate and propionate had anti-inflammatory activity comparable to that of butyrate.

Dürholz et al. (2022) discovered that consuming propionate dramatically decreased megakaryocyte-erythrocyte progenitor (MEP) frequency in the bone marrow of collagen-induced inflammatory arthritis (CIA) models and naïve mice. Despite the following decrease in megakaryocyte numbers, no alterations were seen in usual platelet measures such as platelet counts, mean platelet volume (MPV), and monocyte chemoattractant protein (MCP) levels. However, their observed boosted functional platelet reactivity assays, as well as propionate-induced overall transcriptional activity of platelets and Meg-01 cells, supported previous findings demonstrating that both megakaryocytes and platelets are sensitive to extrinsic influences and can adapt to conditions such as nutritional status and inflammation. Finally, Zhang et al. (2021) found the role of the gut microbiota and SCFAs in Primary Sjögren's syndrome (pSS), suggesting that propionate administration may have a general impact on autoimmune illnesses via megakaryocyte regulation.

Trompette et al. (2014) discovered a method by which dietary fiber content influences the gut microbiota, boosting circulating concentrations of SCFAs that stimulate bone marrow hematopoiesis and decrease DCs' ability to initiate Th2 cell-mediated allergic airway inflammation (AAI). Beyond the close relationship between host cells and bacteria, there is an extra layer of intricacy in which food ingredients and microbial populations affect metabolite accessibility. Trompette et al. (2014) discovered that fermentable fibers in the diet boost the proliferation of bacteria from the *Bacteroidetes* phylum, resulting in higher local and systemic levels of SCFAs, which alter DC hematopoiesis and functioning. Of note, recent research has demonstrated GPR43-mediated growth of colonic Treg cells and found that particular SCFAs might impact Treg cell expansion in either the spleen or the colon (Trompette et al., 2014). In contrast, SCFAs do not lead to an increase in Treg cells in the lung, suggesting that these cells are unlikely to be associated with the impaired ability of lung DCs to sustain allergic inflammation. In summary, the findings show that in inflammatory diseases, the balance between anti-inflammatory microbial metabolites (e.g., SCFAs, IPA) and pro-inflammatory metabolites (e.g., certain SBAs) may determine both disease severity and progression.

### 8.5 Cardiovascular diseases

Although cardiovascular diseases (CVDs) are not the primary focus of hematopoietic regulation, several gut microbial metabolites indirectly link hematopoiesis to CVD pathogenesis through their effects on monocytes, macrophages, and platelets—cells derived from HSCs (Abdalkareem Jasim et al., 2022; Mirzaei et al., 2022). Butyrate reduces monocyte adhesion to endothelial cells by downregulating adhesion molecules such as vascular cell adhesion molecule 1 (VCAM-1) and E-selectin, limiting early vascular inflammation (Wang et al., 2020). In contrast, TMAO, produced via microbial metabolism of dietary choline, has been implicated in atherosclerosis and thrombosis. It promotes foam cell formation by enhancing the expression of cholesterol transporters (e.g., CD36 and ABCA1) in macrophages, and induces a pro-atherogenic phenotype by modulating bile acid synthesis through FXR activation, especially in *ApoE*-deficient mouse models (Li et al., 2018). In mice that are susceptible to atherosclerosis and have low levels of *Apoe*^−/−^, TMAO inhibits the synthesis of bile acids and promotes the formation of aortic lesions via activating FXR and small heterodimer partners (Ding et al., 2018). TMAO's ability to increase the expression of CD36, a scavenger receptor of class A1, and the cholesterol migration-related gene ATP-Binding Cassette Transporter A1 (ABCA1) leads to the buildup of cholesterol in macrophages (Wang et al., 2022). A study conducted by Zhu et al. (2016) showed that the development of atherosclerosis was suppressed in *ApoE*^−/−^ mice that were given a diet containing wild-type substances. The researchers observed that macrophage reverse cholesterol transport (RCT) was promoted in the liver and feces, and there was an increase in ABCA1 expression in the liver (Wang et al., 2022). Research has shown that boosting macrophage RCT is a successful approach to preventing the formation of macrophage-derived foam cells, which is a crucial step in the development of atherosclerosis (Tannahill et al., 2013; Marsch et al., 2014). Zhu et al. (2016) discovered that gut bacteria could influence the possibility of blood clot formation in living organisms.

Dietary choline affects platelet reactivity and function, affecting the overall risk of thrombosis in the circulatory system (Zhu et al., 2016). Choline supplementation specifically led to an increase in the overall activity of choline TMA lyase in the cecum. This rise was accompanied by higher levels of TMAO in the blood and an increased abundance of certain microbial groups associated with TMAO. Zhu et al. (2016) also demonstrated that TMAO has a non-specific impact on platelet hyperresponsiveness. Various substances that stimulate platelets, such as ADP, thrombin, collagen, and arachidonic acid, all exhibited an increase in Ca2+ release from platelet intracellular stores and activation when TMAO was present, even at suboptimal levels of stimulation. Furthermore, research investigating the impact of TMAO on IPA formation in platelets, which is a signaling molecule generated by different phospholipase C isoforms that stimulates the release of intracellular Ca2+ and leads to platelet activation, has also shown an increase in IPA production due to TMAO. While classic risk factors such as hypertension, hypercholesterolemia, and smoking have been extensively studied (Ji et al., 2011), emerging data suggest that the gut microbiota and its metabolites may potentially significantly contribute to the development of CVDs. The relationship between the gut microbiota metabolites and CVD is intricate, as shown by many studies conducted on both humans and animals. Additional study is required to clarify the underlying processes of the effects caused by the introduction of external metabolites or small-molecule therapeutic antagonists, as well as the intricate interconnections between these systems.

### 8.6 Infectious diseases

Microbial metabolites modulate the immune response in various infectious illnesses (Kim, 2018). Carrillo-Salinas et al. (2022) have shown that age-dependent alterations in neutrophil activation, secretory profile, and anti-human immunodeficiency virus (HIV) responses are induced by pathogenic SCFA levels. They observed that different SCFAs had distinct impacts and that older women's neutrophils are more susceptible to SCFA-induced regulation. Carrillo-Salinas et al. (2022) findings are relevant for understanding how neutrophil-mediated innate immune responses in the vaginal tract of younger and older women may be impacted by alterations to the genital microbiota that alter the metabolome. In the current study, they developed an *in vitro* model to assess the possible effects of high-SCFA-concentration environments on neutrophils by administering blood neutrophils with three different SCFAs, which are increased in circumstances with vaginal dysbiosis and at pathological levels (Carrillo-Salinas et al., 2022). CD16 is associated with the lifespan of neutrophils, whereas CD66b indicates adhesion and activation of neutrophils. CD62L is a marker of migration and maturity, which decreases as neutrophils age (Yan et al., 2023). Therefore, the findings of Carrillo-Salinas et al. (2022) indicate that a high level of butyrate is associated with increased lifespan and decreased activation in adult neutrophils that are not aged. Interestingly, the population of CD66^low^ CD16^high^ neutrophils in younger women only showed a slight increase after the administration of butyrate and propionate. However, significant changes in phenotype were mainly observed in older women when SCFAs were present, suggesting that aging may make individuals more susceptible to the effects of SCFAs. The research performed by Carrillo-Salinas et al. (2022), along with other relevant investigations, demonstrates that physiologically normal amounts of SCFAs may function as chemoattractants for immune cells, namely neutrophils. However, when present at excessive levels, SCFAs hinder the movement of neutrophils, regardless of their age (Yan et al., 2023).

A study conducted by Hayashi et al. (2021) demonstrated that the probiotic bacterium CBM588, which generates butyrate, effectively reduces *Clostridium difficile* infection (CDI) by attracting neutrophils and inducing protective T-cell responses (Hayashi et al., 2021). The production of butyrate by CBM588 is very likely to be the source of neutrophil recruitment. Hayashi et al. (2021) discovered that the administration of CBM588 significantly decreased *C. difficile* colonization in mice. This suggests that neutrophils are thought to directly restrict the number of germs present. The neutrophil reduction did not impact the amount of *C. difficile* in feces (Vinolo et al., 2011a). SCFAs might indirectly control neutrophils by stimulating other cell types, such as immune cells, and directly communicating via GPR43. IL-17A enhances the viability and attraction of neutrophils. The increased production of IL-17A by CBM588 may help explain its ability to enhance the Th17 response independently of the butyrate-GPR43 pathway. Their findings demonstrate the impact of the butyrate-producing bacteria CBM588 on neutrophil activity in the gut, resulting in enhanced human defenses against CDI. Curiously, CBM588 is subjected to much lower doses during human clinical trials compared to its administration in animal models. Consequently, the administration of CBM588 may not stimulate a beneficial immune response in a medical setting. Nevertheless, their research provides insight into the causes of acute CDI and uncovers the complexity of the human immune and microbial regulatory systems. Fachi et al. (2020) revealed that acetate therapy impacted CDI in mice, potentially enhancing the advancement of *C. difficile* treatment. These findings demonstrate that acetate produced by microorganisms plays a crucial role in protecting against CDI. Acetate activates FFAR in both neutrophils and ILC3s, resulting in a synchronized increase in the production of IL-1β through inflammasomes in neutrophils and IL-1β responsiveness in ILC3s. Fachi et al. (2020) highlight the significant importance of intestinal microbiota metabolites in treating dysbiosis and gastrointestinal infections, as well as their complex mechanisms of action.

During the process of infection, HSPCs must adjust their production of blood cells to preserve a stable balance and provide an appropriate supply of myeloid cells to fight against pathogens (Galloway-Peña and Jobin, 2023). The activation of TLRs produced from HSCs and HSPCs promotes enhanced cell proliferation, the release of cytokines, and the production of myeloid cells. Activation of the TLR4 or TLR2 pathways has been shown to stimulate the proliferation of HSCs and the differentiation of myeloid cells. Specifically, when exposed to LPS stimulation, myeloid progenitors generate macrophages, whereas lymphoid progenitors make DCs (Nagai et al., 2006). Although it may seem that a brief infection is responsible for the growth, differentiation, and migration of myeloid cells from the bone marrow to the infected tissues, it is interesting to note that acute or chronic TLR signaling may actually hinder the proper function of the HSPCs. For instance, Rodriguez et al. (2009) demonstrated that *P. aeruginosa* infection-induced severe sepsis led to myelosuppression in a way that relies on TLR4 (Galloway-Peña and Jobin, 2023). HSCs derived from mice with sepsis exhibited notable deficiencies in repopulation experiments and could not effectively produce myeloid and granulocyte progenitors. Similarly, animals that were given modest doses of LPS several times could not keep HSPCs in a state of rest, could not produce lymphocytes, and had a bias toward myeloid cells (Galloway-Peña and Jobin, 2023). Aside from LPS, additional microbial elements, including β-glucan from *Candida albicans* cell walls, CpG DNA, and the mycobacterial *Bacillus Calmette-Guerin* (BCG) vaccine, have been demonstrated to stimulate the differentiation of HSPCs, hence facilitating the production of myeloid cells, in a TLR-dependent way (Galloway-Peña and Jobin, 2023). Additionally, it has been shown that other proinflammatory cytokines, such as IL-1, may stimulate the production of myeloid cells in response to infection or inflammation. Finally, Schulthess et al. (2019) showed that butyrate may alter histones in macrophages. This alteration results in increased transcription of antibacterial genes and a better response of macrophages to enteric infections. It has been discovered that macrophages, derived from human blood and induced to differentiate in a laboratory setting using butyrate, had increased effectiveness in suppressing the proliferation of *Salmonella enterica, Serovar typhimurium, E. coli, Staphylococcus aureus*, and *Citrobacter rodentium* (Schulthess et al., 2019). In conclusion, microbial metabolites such as acetate, butyrate, and propionate play a pivotal role in modulating hematologic cell functions during infectious diseases.

## 9 Microbiota metabolites as a therapeutic target in blood cell-associated diseases

The therapeutic implications of microbiota-derived metabolites in hematopoietic disorders should be interpreted cautiously because the majority of mechanistic and intervention studies remain preclinical. Although manipulation of microbial communities, dietary substrates, specific metabolites, microbial biosynthetic pathways, or host receptors has shown beneficial effects in experimental models, these findings do not necessarily establish efficacy in humans. Accordingly, metabolite-based interventions discussed in this review should be considered investigational or hypothesis-generating unless supported by clinical evidence. Translation will require careful assessment of metabolite exposure, tissue distribution, receptor engagement, microbial composition and metabolic capacity, disease stage, treatment context, and potential off-target effects. Clinical studies are therefore needed to determine whether mechanisms demonstrated in experimental models can be safely and reproducibly exploited for therapeutic benefit in patients with hematopoietic disorders. In this regard, emerging evidence highlights the pivotal role of the gut microbiota and its metabolites in shaping the progression and treatment outcomes of hematologic-associated diseases. The gut microbiota contributes to immune system modulation, metabolic regulation, and mucosal barrier integrity—all factors that can significantly influence cancer development and response to therapy (Al-Qadami et al., 2022). In hematologic cancers such as MM, butyrate has been shown to promote Treg expansion while suppressing pro-inflammatory cytokines like IL-17, which is associated with disease progression (Calcinotto et al., 2018). Study by Kusakabe et al. (2020) demonstrated that patients with higher levels of these beneficial bacteria have better post-HSC transplantation (HSCT) survival and fewer complications. A preserved or diverse gut microbiota composition, especially one enriched with SCFA producers, correlates with reduced GVHD, lower infection risk, and overall improved prognosis (Kusakabe et al., 2020; Riwes et al., 2024).

The immunomodulatory properties of microbial metabolites play a vital role in this process, as they can regulate anti-cancer effects, such as the quantity of immunosuppressive Tregs and tumor-killing CD4^+^ and CD8^+^ T cells (Rangan and Mondino, 2022). In addition, butyrate particularly inhibits HDACs, which are associated with regulating cellular proliferation and the cell cycle. Currently, there are ongoing investigations into many HDAC inhibitors as possible medications for treating cancer (Li and Seto, 2016). Various treatment approaches have distinct effects on the gut microbiome, which might possibly lead to complex interactions with microbial metabolites. Furthermore, research has demonstrated that oral administration of butyrate can reduce cardiomyocyte apoptosis and stimulate an anti-inflammatory response by enhancing the polarization of M2 macrophages in the colon, which possess anti-inflammatory properties (Huang et al., 2019). Chen et al. (2022) discovered that the dysbiosis of gut microbiota was linked to cardiotoxicity in an animal model produced by PD-1 and PD-ligand 1 (PD-L1) inhibitors. More precisely, there was a decrease in the prevalence of *Prevotellaceae* and *Rikenellaceae*, which are recognized for their ability to generate SCFAs and facilitate the production of butyrate (Chen et al., 2022). A recent study indicates that butyrate generated by bacteria might mitigate the harmful effects of chemotherapy and immunotherapy on the heart (Chen et al., 2022). This is accomplished by decreasing oxidative stress, reducing ROS and apoptosis in cardiac tissues, as well as reducing inflammatory reactions.

Microbial metabolites might be classified as postbiotics due to their features. These metabolites are produced by probiotic microorganisms and have biological action in the host. However, they are non-viable bacterial metabolic products (Simeoli et al., 2017). The previous study assessed the efficacy of butyrate injection in inhibiting the migration of myeloid and lymphoid cells into the colonic mucosa. It also examined the role of the infiltration of immune cells in the inflammation that occurs during the development of colitis (Tsou et al., 2007). According to these findings, the compound N-(1-carbamoyl-2-phenylethyl) butyramide (FBA) reduced the activity of myeloperoxidase (MPO) and the levels of Ly6g mRNA by inhibiting the migration of PMN cells in the colonic mucosa, which was triggered by dextran sodium sulfate (DSS). Ly6G is a specific marker that solely communicates with neutrophils, making it very useful to recognize cells that come from the neutrophil lineage (Tsou et al., 2007). The inflamed mucosa in individuals with ulcerative colitis is characterized by a significant infiltration of neutrophils and the formation of crypt abscesses (Raab et al., 1993; Shimoyama et al., 2001). Granulocyte adsorption apheresis therapy has been seen to have excellent results in patients with active ulcerative colitis (Shimoyama et al., 2001). Furthermore, the biomarkers calprotectin and lactoferrin, which are generated by neutrophils in feces, are very effective non-invasive assays for detecting inflammatory processes in the gastrointestinal tract (Sipponen, 2013). These findings provide evidence for the essential function of neutrophils in the development of ulcerative colitis. In addition, the movement of PMNs from the small blood vessels to the mucous membrane hinders the barrier's effectiveness and causes tissue damage (Nusrat et al., 1997).

It is well-recognized that uncontrolled inflammatory responses during pneumonia may lead to significant lung damage, impaired function, and an unfavorable prognosis. Galvão et al. (2018) discovered that removing GPR43 resulted in an augmentation of neutrophil influx and endurance, hence contributing to the elevation of IL-1β levels. The production of IL-1β, an important cytokine, plays a crucial role in the immune response to *Klebsiella pneumoniae*. Galvão et al. (2018) noted that neutrophil activities were impaired, leading to a better understanding of the uncontrolled bacterial growth in the airways of *Gpr43*^−/−^ mice. They found that the activation of acetate-GPR43 signaling in inflammatory cells leads to an increase in the production of ROS, activation of the inflammasome, phagocytosis, and the elimination of microorganisms. According to the results obtained by Galvão et al. (2018), the acetate/Gpr43 pathway was discovered to improve the reduction of inflammation in an experimental model and to play a key role in preparing germ-free mice, boosting their ability to respond to a sterile injury.

The growing appreciation of gut microbiota-derived metabolites in regulating hematopoietic cell development has prompted significant interest in leveraging microbiota-targeted interventions as a therapeutic strategy (Hitch et al., 2022). These interventions—ranging from dietary modulation and probiotics to postbiotics and fecal microbiota transplantation (FMT)—aim to restore or enhance beneficial microbial functions and thereby influence hematopoietic homeostasis or disease outcomes (Hitch et al., 2022; Guo et al., 2024). However, translating these strategies into clinical practice is fraught with several challenges that merit consideration. One major limitation is the strain-specific effect of probiotic organisms (Mejía-Caballero et al., 2021). Even within a single species, such as *Lactobacillus rhamnosus* or *Bifidobacterium longum*, strains can vary significantly in their capacity to colonize the gut, produce bioactive metabolites (e.g., SCFAs or indole derivatives), and interact with host immune cells (Mafe et al., 2025). This heterogeneity complicates the standardization of probiotic therapies and may contribute to inconsistent clinical outcomes (Schupack et al., 2022). Moreover, the host's baseline microbiota composition can influence colonization resistance, further adding to variability in treatment response.

Another critical consideration is the bioavailability and distribution of microbial metabolites (Zhang et al., 2020). While compounds like butyrate, propionate, or tryptophan catabolites can exert potent immunomodulatory effects *in vitro* and in murine models, their physiological concentrations in specific niches such as the bone marrow or thymus remain unclear (Belkaid and Harrison, 2017). Many microbial metabolites are rapidly absorbed, degraded, or metabolized in the gut, which limits their systemic availability and therapeutic efficacy (Belkaid and Harrison, 2017). Hence, strategies to enhance metabolite stability or develop targeted delivery systems—such as encapsulation techniques or engineered microbes—are actively being explored (Belkaid and Harrison, 2017; Mejía-Caballero et al., 2021). However, while microbiota-targeted therapies hold transformative potential for modulating hematopoiesis and treating hematologic disorders, their clinical translation requires careful consideration of microbial ecology, metabolite pharmacokinetics, and personalized host-microbiome interactions (Wang et al., 2024).

## 10 Translational relevance and limitations of experimental evidence

Although experimental studies have provided substantial mechanistic insight into microbiota–metabolite regulation of hematopoiesis, the available evidence should be interpreted according to the experimental system in which it was generated. A considerable proportion of mechanistic data concerning direct effects of microbial metabolites on HSPCs, hematopoietic lineage commitment, and bone-marrow niche remodeling originates from mouse or other animal models and *in vitro* systems. These studies are essential for establishing causality and identifying molecular pathways but do not necessarily demonstrate that identical mechanisms operate quantitatively or qualitatively in humans. For example, experimental evidence indicates that AhR activation by microbial metabolites can alter HSC proliferation and lineage bias, whereas loss of AhR signaling can promote HSC exhaustion; however, the extent to which this pathway produces the same cellular effects in human HSCs remains incompletely defined (Singh K. P. et al., 2014; Seo et al., 2025). In contrast, several aspects of the microbiota–hematopoiesis relationship have been supported by human clinical or observational evidence. Reduced intestinal microbial diversity in patients undergoing allogeneic hematopoietic stem cell transplantation is associated with poorer clinical outcomes and mortality, supporting the clinical relevance of microbiome disruption in hematopoietic transplantation (Taur et al., 2014; Skaarud et al., 2021). Similarly, clinical observations indicate that dietary fiber intake can increase hemoglobin levels in individuals with anemia and can be accompanied by increased SCFA, particularly butyrate, concentrations in patients with renal anemia (Li et al., 2022). These findings support a relationship between diet, microbial metabolism, and hematological status, but they do not establish that the observed hematological effects are mediated exclusively by SCFAs. Human studies have also provided evidence for metabolite-related hematological effects in specific disease settings. For example, butyrate-related interventions have been investigated in individuals with hemoglobinopathies, including studies examining γ-globin and fetal hemoglobin induction (Weinberg et al., 2005). Nevertheless, the presence of a clinical response in selected patients should not be interpreted as definitive evidence that the endogenous microbiota-derived butyrate pathway regulates hematopoiesis through the same mechanisms demonstrated experimentally. Accordingly, it is important to distinguish clinical association, experimental causality, and translational validation. Associations between microbial taxa or circulating metabolites and disease outcomes in humans provide evidence of clinical relevance but cannot independently establish causality. Conversely, animal and cellular models can demonstrate causal relationships and identify receptor-, pathway-, or cell-specific mechanisms, but their findings require confirmation in human systems. Several mechanisms discussed in this review should therefore currently be regarded as preclinical or hypothesis-generating rather than clinically established. This is particularly true for proposed direct effects of specific metabolites on abnormal HSPCs and for metabolite-mediated remodeling of the bone-marrow niche in diseases such as myelodysplastic syndromes, where the causal contribution of individual microbial metabolites remains insufficiently defined. Future studies should prioritize longitudinal human cohorts, quantitative metabolomics, functional microbiome profiling, *ex vivo* studies using human HSPCs and bone-marrow niche cells, and prospective clinical trials to determine whether experimentally defined pathways are reproducible in patients. Thus, the current evidence supports a model in which the biological plausibility and mechanistic framework are strongly supported by experimental research, whereas the clinical relevance of several pathways is supported by human associations and selected clinical observations but remains incompletely validated at the mechanistic level. Explicitly separating these evidence levels is essential for translating microbiota-derived metabolite biology into reliable therapeutic strategies.

## 11 Conclusion and future directions

The interactions of microbiota metabolites and hematopoiesis have been identified as an interesting topic to study in terms of immune homeostasis regulation and hematopoietic function. Recent findings show that bacterial metabolites play a direct part in the regulation of the hematopoietic system by regulating proliferation, differentiation, and functions of immune cells. The increase in SCFA production caused by microbial fermentation due to dietary fiber promotes the generation of DC precursors in the bone marrow; however, at the same time, it lowers their ability to induce a Th2 reaction, thus preventing allergic airway inflammation. Moreover, SCFAs, for example, propionate, can be considered a therapeutic agent in autoimmune diseases such as systemic lupus erythematosus via modulation of megakaryocyte functions and regulation of inflammatory cytokine production. Besides the influence on hematopoiesis and inflammatory diseases, recent data highlight the involvement of microbiota metabolites in the regulation of anticancer immunity. Moreover, indoles and IPA, as metabolic products of the gut microbiota, contribute to the enhancement of cytotoxicity of γδ T cells that can be used for improvement of cancer immunotherapy. Thus, the existing data reveal the fundamental role of bacterial metabolites in the regulation of hematopoietic and immune system processes. Further investigation of the interaction between microbiota and hematopoiesis can help understand the molecular background of the interaction and identify new targets for drug development.

Of note, the complexity of the microbiota–metabolite–hematopoiesis axis necessitates approaches capable of resolving cellular, spatial, metabolic, and microbial heterogeneity simultaneously. Conventional microbiome profiling can identify associations between microbial taxa and hematological phenotypes, but taxonomic abundance alone cannot determine which microbial functions are active, which metabolites are produced, or which hematopoietic and niche cells respond to these signals. Emerging single-cell and multi-omics technologies therefore offer an important opportunity to move from descriptive associations toward cell-specific and mechanistically resolved models of microbiota–hematopoiesis communication. Single-cell RNA sequencing (scRNA-seq) and related single-cell multi-omic approaches could identify heterogeneous responses among HSPCs and mature hematopoietic populations following changes in microbial metabolites. By resolving transcriptional states at single-cell resolution, these approaches may reveal metabolite-responsive cellular subsets, lineage-specific signaling programs, and changes in differentiation trajectories that are obscured in bulk tissue analyses. This is particularly relevant because microbial metabolites can influence hematopoiesis both directly through HSPCs and indirectly through stromal, endothelial, macrophage, and other niche populations.

Metabolomics provides a complementary functional layer by enabling quantitative characterization of circulating and tissue-associated microbial metabolites. Measurement of SCFAs, tryptophan derivatives, bile acids, TMAO, lactate, and other microbial products alongside hematopoietic phenotypes could determine whether changes in microbial abundance are accompanied by corresponding alterations in metabolic output. This distinction is critical because dysbiosis can involve both depletion of potentially protective metabolites and accumulation of inflammatory microbial products. Integration of microbiome multi-omics with host molecular profiling could provide an even more comprehensive framework. Shotgun metagenomics can characterize the genetic potential of microbial communities, whereas metatranscriptomics and metabolomics can provide information regarding microbial pathway activity and actual metabolic output. When these datasets are integrated with single-cell transcriptomic, epigenomic, and clinical data, it may become possible to construct mechanistic links between a microbial pathway, its metabolic product, the host receptor or signaling pathway activated, and the specific hematopoietic or niche cell affected. These approaches may distinguish conserved mechanisms from patient-specific responses, identify metabolic pathways that are causally linked to hematopoietic dysfunction, and reveal biomarkers capable of predicting therapeutic response. Such an integrated strategy could shift the field from identifying “which bacteria are associated with disease” toward determining which microbial functions and metabolites act on which cells, within which anatomical niches, at which stages of disease, and with what biological consequences. This level of resolution will be essential for translating microbiota-derived metabolite biology into precise therapeutic strategies.
